# Distinct *HLA* associations with autoantibody-defined subgroups in idiopathic inflammatory myopathies

**DOI:** 10.1016/j.ebiom.2023.104804

**Published:** 2023-09-26

**Authors:** Valérie Leclair, Angeles S. Galindo-Feria, Simon Rothwell, Olga Kryštůfková, Sepehr Sarrafzadeh Zargar, Herman Mann, Louise Pyndt Diederichsen, Helena Andersson, Martin Klein, Sarah Tansley, Lars Rönnblom, Kerstin Lindblad-Toh, Ann-Christine Syvänen, Marie Wahren-Herlenius, Johanna K. Sandling, The Dissect Consortium and The Immunoarray Development Consortium, Matteo Bianchi, Sergey V. Kozyrev, Johanna K. Sandling, Lars Rönnblom, Maija-Leena Eloranta, Ann-Christine Syvänen, Dag Leonard, Johanna Dahlqvist, Maria Lidén, Argyri Mathioudaki, Jennifer RS. Meadows, Jessika Nordin, Gunnel Nordmark, Ingrid E. Lundberg, Antonella Notarnicola, Leonid Padyukov, Anna Tjärnlund, Maryam Dastmalchi, Daniel Eriksson, Øyvind Molberg, Helena Andersson, Kerstin Lindblad-Toh, Fabiana H.G. Farias, Marie Wahren-Herlenius, Awat Jalal, Balsam Hanna, Helena Hellström, Tomas Husmark, Åsa Häggström, Anna Svärd, Thomas Skogh, Louise Pyndt Diederichsen, Janine A. Lamb, Simon Rothwell, Hector Chinoy, Robert G. Cooper, Kerstin Lindblad-Toh, Gerli Rosengren Pielberg, Anna Lobell, Åsa Karlsson, Eva Murén, Kerstin M. Ahlgren, Lars Rönnblom, Maija-Leena Eloranta, Göran Andersson, Nils Landegren, Olle Kämpe, Peter Söderkvis, Neil McHugh, Janine A. Lamb, Jiri Vencovský, Hector Chinoy, Marie Holmqvist, Matteo Bianchi, Leonid Padyukov, Ingrid E. Lundberg, Lina-Marcela Diaz-Gallo

**Affiliations:** a Clinical Epidemiology Division, Department Medicine Solna, Karolinska Institutet, Karolinska University Hospital, Stockholm, Sweden; b Division of Rheumatology, Jewish General Hospital Lady Davis Institute, Montreal, Canada; c Division of Rheumatology, Department of Medicine, Solna, Karolinska Institutet and Karolinska University Hospital, Stockholm, Sweden; d Center for Molecular Medicine, Karolinska Institutet and Karolinska University Hospital, Stockholm, Sweden; e Centre for Genetics and Genomics Versus Arthritis, Centre for Musculoskeletal Research, Faculty of Biology, Medicine and Health, University of Manchester, Manchester, United Kingdom; f Institute of Rheumatology and Department of Rheumatology, 1st Medical Faculty, Charles University, Prague, Czech Republic; g Center for Rheumatology and Spine Diseases, Copenhagen University Hospital, Rigshospitalet, Copenhagen, Denmark; h Department of Rheumatology, Odense University Hospital, Odense, Denmark; i Department of Rheumatology, Oslo University Hospital, Oslo, Norway; j Department of Life Sciences, University of Bath, Bath, United Kingdom; k Department of Medical Sciences, Rheumatology, Uppsala University, Uppsala, Sweden; l Science for Life Laboratory, Department of Medical Biochemistry and Microbiology, Uppsala University, Uppsala, Sweden; m Broad Institute of MIT and Harvard, Cambridge, MA, Unite States of America; n Science for Life Laboratory, Uppsala University, Department of Medical Sciences, Molecular Precision Medicine, Uppsala, Sweden; o Broegelmann Research Laboratory, Department of Clinical Science, University of Bergen, Norway; p Epidemiology and Public Health Group, Faculty of Biology, Medicine and Health, University of Manchester, United Kingdom; q Department of Rheumatology, Salford Royal Hospital, Northern Care Alliance NHS Foundation Trust, Manchester Academic Health Science Centre, Salford, United Kingdom; r Division of Musculoskeletal and Dermatological Sciences, Faculty of Biology, Medicine and Health, The University of Manchester, Manchester, United Kingdom

**Keywords:** Autoantibody, *HLA*, Idiopathic inflammatory myopathy, Myositis

## Abstract

**Background:**

In patients with idiopathic inflammatory myopathies (IIM), autoantibodies are associated with specific clinical phenotypes suggesting a pathogenic role of adaptive immunity. We explored if autoantibody profiles are associated with specific *HLA* genetic variants and clinical manifestations in IIM.

**Methods:**

We included 1348 IIM patients and determined the occurrence of 14 myositis-specific or –associated autoantibodies. We used unsupervised cluster analysis to identify autoantibody-defined subgroups and logistic regression to estimate associations with clinical manifestations, *HLA-DRB1, HLA*-*DQA1, HLA-DQB1* alleles, and amino acids imputed from genetic information of HLA class II and I molecules.

**Findings:**

We identified eight subgroups with the following dominant autoantibodies: anti-Ro52, -U1RNP, -PM/Scl, -Mi2, -Jo1, -Jo1/Ro52, -TIF1γ or negative for all analysed autoantibodies. Associations with *HLA-DRB1∗11, HLA-DRB1∗15, HLA-DQA1∗03,* and *HLA-DQB1∗03* were present in the anti-U1RNP-dominated subgroup. *HLA-DRB1∗03, HLA-DQA1∗05,* and *HLA-DQB1∗02* alleles were overrepresented in the anti-PM/Scl and anti-Jo1/Ro52-dominated subgroups. *HLA*-*DRB1∗16*, *HLA-DRB1∗07* alleles were most frequent in anti-Mi2 and *HLA*-*DRB1∗01* and *HLA-DRB1∗07* alleles in the anti-TIF1γ subgroup. The *HLA-DRB1∗13, HLA-DQA1∗01* and *HLA-DQB1∗06* alleles were overrepresented in the negative subgroup. Significant signals from variations in class I molecules were detected in the subgroups dominated by anti-Mi2, anti-Jo1/Ro52, anti-TIF1γ, and the negative subgroup.

**Interpretation:**

Distinct *HLA* class II and I associations were observed for almost all autoantibody-defined subgroups. The associations support autoantibody profiles use for classifying IIM which would likely reflect underlying pathogenic mechanisms better than classifications based on clinical symptoms and/or histopathological features.

**Funding:**

See a detailed list of funding bodies in the Acknowledgements section at the end of the manuscript.

**Research in context**

**Evidence before this study**

*HLA* associations with different autoantibody specificities in idiopathic inflammatory myopathies (IIM) have been studied before. However, no studies have considered the overlap of multiple myositis-specific and –associated autoantibodies in the context of genetic variations in the *HLA* region using DNA-based variabilities and imputed amino acids.

**Added value of this study**

Our results demonstrate distinctive *HLA* genetic associations with subgroups of patients with idiopathic inflammatory myopathies, defined by both myositis-specific and –associated autoantibodies.

**Implications of all the available evidence**

The combination of *HLA* genotypes and autoantibody profiles, including myositis-specific and –associated autoantibodies, offers a different perspective on IIM subgrouping and the pathogenic mechanisms underlying these subgroups.

## Introduction

Idiopathic inflammatory myopathies (IIM) are rare autoimmune multisystemic diseases. Major IIM subsets are dermatomyositis (DM), juvenile DM, anti-synthetase syndrome, immune-mediated necrotizing myositis (IMNM), polymyositis (PM), and inclusion body myositis (IBM).^1^^,^^2^ Autoantibody discovery in IIM has improved diagnosis, clinical phenotyping, management, and understanding of the disease's molecular mechanisms. Among the sixteen myositis-specific autoantibodies (MSA) commonly screened for in the clinic (i.e., anti-Jo1, -PL7, -PL12, -OJ, -EJ, -KS, -Zo, -HA, -SRP, -HMGCR, -Mi2, -MDA5, -NXP2, -SAE, -TIF1y/α, -cN1A), anti-histidyl-transfer RNA synthetase (anti-Jo1) is found in ∼20% of adult-onset patients, while most other autoantibodies are relatively rare.^3^ While MSAs are usually monospecific (i.e., only one MSA is present in an individual), they may be found in combination with myositis-associated autoantibodies (MAA). Anti-Ro52/TRIM21, -U1RNP, -Ku and -PM/Scl are MAA that can be found in combination with MSA, although they can also be detected in isolation.^3^ The presence of anti-Ro52/TRIM21 in patients with anti-MDA5 antibodies or anti-synthetase syndrome is reported as a marker of poor prognosis.^4^^,^^5^ However, it is unclear if the presence of combinations of MSA and MAA is associated with distinct pathogenic mechanisms.

Genetic variations within the human leukocyte antigen (*HLA*) locus constitute the strongest known genetic risk factors for IIM.^6^ The *HLA* genes encode the class II and I antigen-presenting molecules with primary functions to present short peptides to T-cells. This intercellular interaction can initiate antigen-specific adaptive immune responses, sometimes resulting in the production of autoantibodies. In IIM, different *HLA* alleles are associated with specific autoantibodies.^7^, ^8^, ^9^, ^10^ However, previous studies in IIM have only considered the associations between isolated MSA/MAA and different *HLA* alleles, disregarding multiple specificities. Additionally, no sufficiently large study has investigated the relationships between *HLA* alleles, autoantibody patterns, and clinical/histopathological features to determine which adaptive immune mechanisms are of pathogenic importance in various IIM subgroups.

Different autoantibody profiles (MSA and/or MAA) may reflect disease mechanisms genetically driven by *HLA* alleles. Therefore, we used a large international IIM cohort to identify autoantibody-defined subgroups based on MSA/MAA status allowing for multiple specificities in the same patient if present. This enabled us to investigate the relationship between autoantibody profiles, clinical manifestations, genotyped and imputed *HLA-DRB1, HLA-DQA1, HLA-DQB1* alleles, and imputed class II and I amino acids.

## Methods

### Study population

The MYONET registry was used to identify patients with IIM in clinical registries from five European countries: UK Myositis Network (UKMYONET) (United Kingdom (UK)),^11^ Institute of Rheumatology (Prague, Czech Republic), Karolinska University Hospital (Stockholm, Sweden), Odense and Copenhagen University Hospitals (Odense and Copenhagen, Denmark) and Oslo University Hospital (Oslo, Norway)). We included patients if they: 1) were adults (>18 years old) with at least a possible IIM diagnosis per the 2017 EULAR/ACR classification criteria, 2) had available basic clinical features, and 3) had complete autoantibody profiles (see *Autoantibody testing*). Anti-synthetase syndrome was defined as anti-tRNA synthetase positivity and ⩾1 of: myositis, Raynaud's phenomenon, arthritis, interstitial lung disease (ILD), fever or mechanic's hands.^12^ Overlap myositis (OM) was defined as DM or PM diagnosed in the presence of another connective tissue disease.

### Patient and public involvement

Patients’ representatives and the public were not involved in the planning and conduction of this study.

### Clinical features

Clinical features were assessed as present or absent at any time point during follow-up as defined by the MYONET registry definitions (Supplementary Table S1).^13^ Sex was self-reported by study participants. Myopathic muscle weakness was defined as objective subacute symmetric muscle weakness, proximal more than distal, sparing the eye and facial muscles. ILD was considered present if described on a chest radiograph or computed tomography with abnormal pulmonary function testing. DM rashes included the presence of heliotrope rash and Gottron's papules or signs. The treating physician assessed the presence of ulceration, calcinosis, Raynaud's phenomenon, and arthritis. Dysphagia was defined as difficulty in swallowing or objective evidence of abnormal esophagus motility.

### Autoantibody testing

Autoantibodies against Jo1, PL12, PL7, OJ, EJ, Mi2, MDA5, NXP2, TIF1γ, SAE1, SRP, PM/Scl, Ro52, and U1RNP were analysed in each centre using one or more of 1) immunoprecipitation, 2) Euroline myositis panel 3 or 4 by Euroimmun, Lübeck, Germany, and/or 3) enzyme-linked immunosorbent assays. If more than one method was available per patient and results were discordant, the screening time point closer to diagnosis was kept giving precedence to immunoprecipitation if available.^14^ See Supplementary Table S2 for detailed methods used by the different recruiting centres.

### HLA alleles and amino acid determination

*HLA-DRB1, HLA-DQA1,* and *HLA-DQB1* alleles were directly genotyped by sequence-specific primer PCR assay, microarray, or multiplex assay, using the genomic DNA (Supplementary Table S2). To explore association signals from *HLA* class I (*HLA-B*, -*C* and *-A*) and the autoantibody-defined subgroups, we imputed amino acids from genotyping information available from the Dissect Consortium (Supplementary Information 1) for the UK and Scandinavia (i.e., Sweden, Denmark, and Norway) patients. Since this represents a smaller sample size, we tested only for amino acids (Supplementary Information 2), which encompasses the genetic variation of both *HLA* class II and I while reducing the multiple comparison burden.

### Statistical analysis

Descriptive statistics were used to summarize the baseline characteristics of the study population. An unsupervised cluster analysis included patients with at least one positive autoantibody. Patients negative for all the included autoantibodies were considered a distinct subgroup. Autoantibody-based subgroups were created using the Gower distance matrix and partition around medoids cluster calculation.^15^^,^^16^ Each cluster was labelled using a medoid representing the individual in the subgroup that yielded the lowest average distance. The number of subgroups was selected considering the Silhouette metric (Supplementary Figure S1), and the sample size per subgroup. Kruskal–Wallis test was used to compare the median age between the subgroups. Logistic regression adjusted for age and sex was used to estimate the p-value, odds ratios (OR), and 95% confidence intervals (CI) of having specific clinical manifestations, *HLA-DRB1, HLA*-*DQA1, HLA-DQB1* alleles, and imputed amino acids frequencies depending on the subgroup assigned. In the logistic regression for the clinical associations, the outcome was having or not a given clinical feature, while the independent variable was either being in one of the individual subgroups (e.g., subgroup 1) versus being in the rest of the cohort. These models were adjusted for sex, age as a quadratic term, and recruiting centre. For the *HLA* associations, the dependent variable was the subgroup assignment, and the independent variable was the different HLA genetic variants controlled by sex and age. The models for the genetic associations were applied separately by regions and subsequently meta-analysed. The Cochran-Mantel-Haenszel test was used to calculate the OR (95% CI) for the meta-analyses and heterogeneity calculated using Cochran's Q test and Higgins' test (I^2^). Given the low heterogeneity across regions, the pooled ORs were estimated using fixed-effect models. The genetic association p-values were adjusted by the Benjamini & Yekutieli step-up method for false discovery rate (FDR).^17^ We considered significant results at a 5% FDR. Geographical regions were divided into the UK, Scandinavia (Sweden, Denmark, and Norway), and the Czech Republic. To determine whether the *HLA* association signals were independent, we performed logistic regression conditioning by the highest associated variants, including sex, age, and population as covariates. The genetic association analyses were performed using PLINK (version 1.9).^18^ All other statistical analyses were performed using R versions 3.6.1 and 4.1.1^19^ (details in Supplementary Information 3).

### Ethics

Use of data from each clinical registry received ethical approval from the relevant local ethical committees (Czech Republic: 3233/2007, Denmark: S-20100022, Norway: 2011/17,553, 2010/2970, 2016/119, UK, 98/8/086, Sweden: Dnr 2023-00244-02, 2012/736-32, 2015/450) and all patients gave informed consent to participate in this study. The ethical approvals were all in agreement with the Helsinki declaration for clinical studies.

### Role of funders

None of the funding sources were involved in the study design, data collection, data analyses, interpretation, or writing the manuscript.

## Results

Complete autoantibody profiles were available for 1348 IIM patients. The median [IQR] age at diagnosis was 55 [42–65], and 65% were female (Table 1). The median age at diagnosis was significantly different between the groups (p < 0.001, Kruskal–Wallis) subgroup 5 having the youngest age at diagnosis (48 [95% CI 35–58]) and subgroup 8 the oldest (58 [95% CI 46–68]). When stratified by individual centre, 54% were from the UK, 31% from Scandinavia and 16% from the Czech Republic. The different centres had similar distributions of IIM subsets except for an increase in the prevalence of DM in the Czech Republic and PM in Denmark. Few overlap myositis and IBM cases were reported in the Czech Republic, Denmark, and Norway (Supplementary Table S3). The UK cohort had the highest number of patients negative for all autoantibodies analysed (58%), followed by Denmark (47%), Sweden (36%), the Czech Republic (30%), and Norway (26%). Regarding the total cohort, 519 patients (39%) were negative for all autoantibodies analysed, leaving 829 (61%) patients for inclusion in the cluster analysis.

**Table 1:** Cohort characteristics stratified by subgroups.

Medoid	Subgroups								All
	1	2	3	4	5	6	7	8	
	Anti-Ro52	Anti-U1RNP	Anti-PM/Scl	Anti-Mi2	Anti-Jo1	Anti-Jo1/Ro52	Anti-TIF1γ	None^a^	
**n (%)**	137 (10)	183 (14)	107 (8)	65 (5)	119 (9)	140 (10)	78 (6)	519 (39)	1348 (100)
**Female,** n (%)	93 (68)	116 (63)	79 (74)	45 (69)	76 (64)	96 (69)	64 (82)	313 (60)	882 (65)
**Age at diagnosis,** median [IQR]	56 [48–64]	52 [39–62]	51 [38–63]	57 [47–69]	48 [35–58]	52 [40–60]	54 [43–65]	58 [46–68]	55 [42–65]
**Center,** n (%)									
UK	56 (41)	113 (62)	60 (56)	37 (57)	64 (54)	58 (41)	34 (44)	302 (58)	724 (54)
Sweden	34 (25)	45 (25)	22 (21)	7 (11)	28 (24)	26 (19)	27 (35)	107 (21)	296 (22)
Czech Republic	28 (20)	13 (7)	19 (18)	17 (26)	19 (16)	36 (26)	16 (21)	62 (12)	210 (16)
Denmark	13 (10)	6 (3)	4 (4)	0	6 (5)	15 (11)	0	39 (8)	83 (6)
Norway	6 (4)	6 (3)	2 (2)	4 (6)	2 (2)	5 (4)	1 (1)	9 (2)	35 (3)
**Clinical features,** n (%)									
Weakness	120 (88)	170 (93)	90 (84)	59 (91)	108 (91)	130 (93)	70 (90)	480 (93)	1227 (91)
ILD	50 (37)	48 (26)	44 (41)	7 (11)	84 (71)	109 (78)	7 (9)	58 (11)	407 (30)
Mechanic′s hands	23 (17)	20 (11)	46 (43)	14 (22)	32 (27)	52 (37)	17 (22)	27 (5)	231 (17)
Gottron′s	31 (23)	57 (31)	44 (41)	43 (66)	27 (23)	27 (19)	63 (81)	89 (17)	381 (28)
Heliotrope	37 (27)	57 (31)	33 (31)	42 (65)	22 (19)	12 (9)	59 (76)	86 (17)	348 (26)
Ulceration	10 (7)	11 (6)	2 (2)	4 (5)	1 (1)	2 (1)	7 (9)	4 (1)	40 (3)
Calcinosis	6 (4)	7 (4)	8 (8)	0	0	3 (2)	6 (8)	12 (2)	42 (3)
Raynaud	37 (27)	74 (40)	51 (48)	14 (22)	37 (31)	53 (38)	10 (13)	82 (16)	358 (27)
Arthritis	33 (24)	60 (33)	29 (27)	14 (22)	65 (55)	83 (59)	10 (13)	101 (20)	395 (29)
Dysphagia	58 (42)	78 (43)	52 (49)	31 (48)	26 (22)	39 (28)	41 (53)	191 (37)	516 (38)
**Autoantibody,** n (%)									
Anti-Jo1	0	6 (3)	0	1 (2)	119 (100)	140 (100)	0	0	266 (20)
Anti-PL7	7 (5)	13 (7)	0	0	0	0	0	0	20 (2)
Anti-PL12	5 (4)	3 (2)	1 (1)	0	1 (1)	0	0	0	10 (1)
Anti-EJ	2 (2)	0	0	0	0	0	0	0	2 (1)
Anti-OJ	0	7 (4)	0	0	0	0	0	0	7 (1)
Anti-TIF1γ	10 (7)	2 (1)	2 (2)	0	0	0	78 (100)	0	92 (7)
Anti-Mi2	1 (1)	1 (1)	1 (1)	65 (100)	0	2 (1)	0	0	70 (5)
Anti-SAE1	8 (6)	23 (13)	0	0	0	0	0	0	31 (2)
Anti-NXP2	1 (1)	23 (13)	1 (1)	0	0	0	0	0	25 (2)
Anti-MDA5	9 (7)	10 (6)	1 (1)	1 (2)	0	1 (1)	0	0	22 (2)
Anti-SRP	8 (6)	32 (18)	0	0	0	0	0	0	40 (3)
Anti-Ro52	137 (100)	16 (9)	0	0	0	140 (100)	0	0	293 (22)
Anti-PMScl	11 (8)	1 (1)	107 (100)	0	0	0	0	0	119 (9)
Anti-U1RNP	0	79 (43)	0	0	0	3 (2)	0	0	82 (6)
**IIM subsets,** n (%)									
DM	40 (29)	59 (32)	38 (36)	61 (94)	0	0	71 (91)	114 (22)	383 (28)
PM	32 (23)	31 (17)	22 (21)	1 (2)	0	0	2 (3)	181 (35)	269 (20)
ASyS	17 (12)	26 (14)	5 (5)	0	118 (99)	140 (100)	1 (1)	10 (2)	317 (24)
OM	24 (18)	49 (27)	35 (33)	1 (2)	0	0	2 (3)	52 (10)	163 (12)
IBM	19 (14)	2 (1)	4 (4)	1 (2)	0	0	1 (1)	119 (23)	146 (11)
IMNM	3 (2)	12 (7)	0	0	1 (1)	0	0	34 (7)	50 (4)
JDM	1 (1)	2 (1)	3 (3)	1 (2)	0	0	1 (1)	8 (2)	16 (1)
Unspecific	1 (1)	2 (1)	0	0	0	0	0	1 (0)	4 (0)

Legend: ILD, interstitial lung disease; DM, dermatomyositis; PM, polymyositis; ASyS, anti-synthetase syndrome; OM, overlap myositis; IBM, inclusion body myositis; IMNM, Immune-mediated necrotising myositis; JDM, juvenile dermatomyositis.

a None of the autoantibodies screened for in this study.

### Overall genetic and clinical associations with serologically defined IIM subgroups

**Fig. 1:**
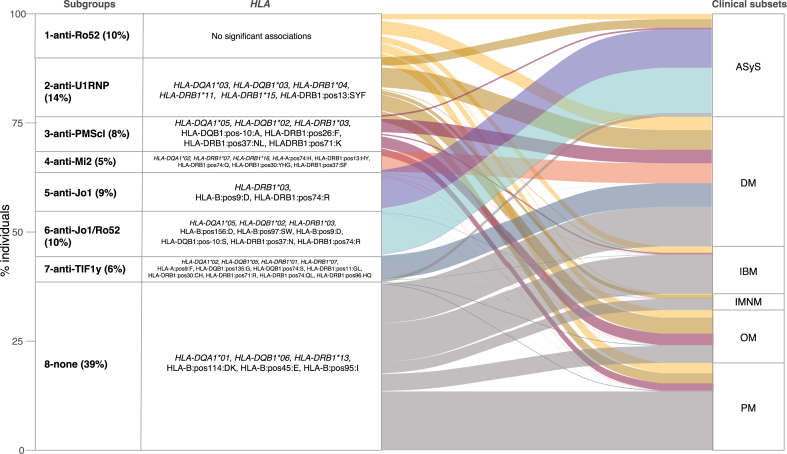
**IIM subgroups based on autoantibody profiles, *HLA* significant associations, and their correspondence to the clinical/pathological subsets**. Distribution of the subgroups defined by autoantibody profiles, their *HLA* associated alleles, amino acids, and clinical/pathological subsets. Clinical/pathological subsets were defined using the EULAR/ACR classification criteria,^1^ the *Connors* et al.^12^ definition for ASyS and the presence of another connective tissue disease for OM. In the representation of the HLA amino acids, the first term is the protein, the second is the position, and the third is the amino acid. Only the positive HLA associations are shown. ASyS, anti-synthetase syndrome; IBM, inclusion body myositis; JDM, juvenile dermatomyositis; PM, polymyositis; DM, dermatomyositis; IMNM, Immune-mediated necrotising myositis; OM, overlap myositis. The unspecific clinical subset (UNS) was not included in this figure, given the very low number of patients (n = 4). The JDM subset is included in the DM clinical subset.

**Fig. 2:**
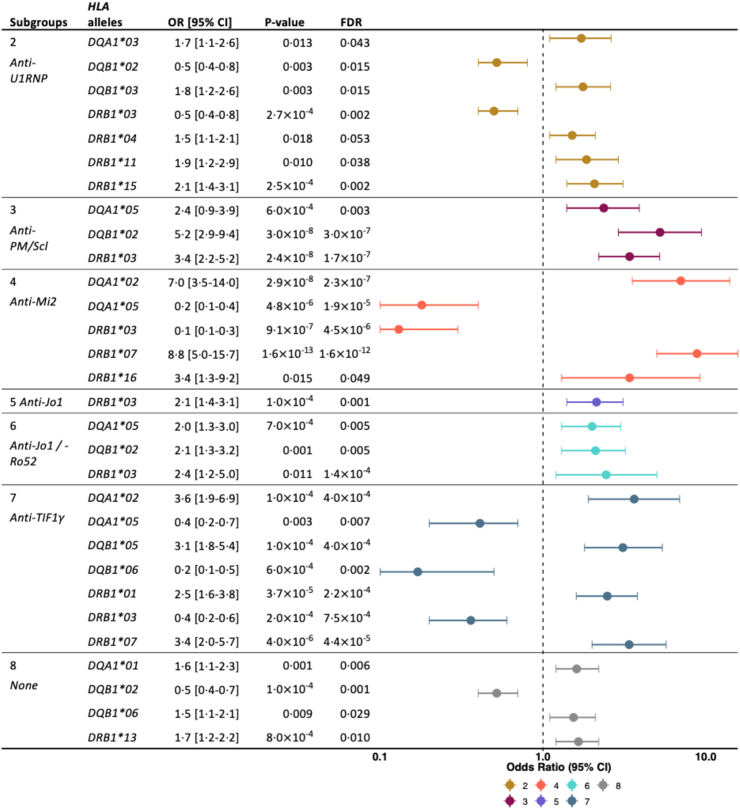
**Significant associations of *HLA-DQA1, HLA-DQB1,* and *HLA*-*DRB1* alleles with autoantibody-defined subgroups**. Significant results from the meta-analyses of the different geographical regions (Czech Republic, Scandinavia, UK). Each subgroup was compared to the rest of the cohort. OR, odds ratio; CI, confidence interval; FDR, false discovery rate.

**Fig. 3:**
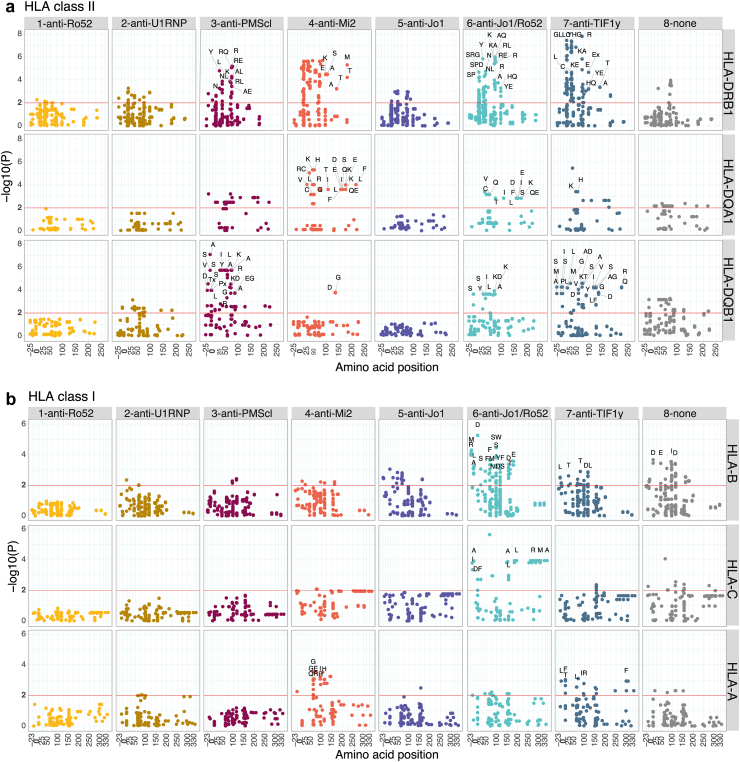
**Significant associations of *HLA* amino acids with autoantibody-defined IIM subgroups**. Manhattan-plot of the meta-analyses of UK and Scandinavia from the imputed amino acids. (**a and b**) Subgroups in columns and genes in rows. Some amino acids with a FDR<0.01 are labelled, and the red line indicates the significance threshold of 5% FDR (**a***:* Class II; **b**: Class I). A, alanine; C, cysteine; D, aspartic acid; E, glutamic acid; F, phenylalanine; G, glycine; H, histidine; I, isoleucine; K, lysine; L, leucine; M, methionine; N, asparagine; P, proline; Q, glutamine; S, serine; T, threonine; R, arginine; V, valine; W, tryptophan; Y, tyrosine; OR, odds ratio.

In addition to the subgroup of patients negative for all autoantibodies analysed, we selected seven autoantibody-defined subgroups (Table 1, Supplementary Figure S1). Three subgroups (1, 2 and 6) were heterogeneous according to their serological profiles, while the other subgroups were mainly defined by the presence of one specific autoantibody (Table 1). Significant and differential associations between the serologically defined subgroups and *HLA* variants were seen for almost all the subgroups compared to the rest of the cohort (Fig. 1, Fig. 2, Fig. 3 and Supplementary Tables S4 and S5). The genetic associations stratified by regions (i.e., UK, Scandinavia, and the Czech Republic) were generally in the same direction, implying low inconsistency and variation (Q and I^2^ tests, Supplementary Table S4).

The description of each serologically defined subgroup is presented in Table 1. Fig. 1 summarizes the overall genetic associations for the serologically defined subgroups in relation to clinical subsets. The DM, PM, and OM clinical subsets were dispersed in several subgroups, while anti-synthetase syndrome, IBM, and IMNM grouped more homogeneously. Notably, there was a higher concordance between the serologically defined subgroups and clinical subsets partially defined by serology, such as the anti-synthetase syndrome, compared to clinically and histopathologically defined subsets, such as DM, PM, and OM. Table 2 presents the clinical feature associations for each subgroup.

**Table 2:** Associations between clinical manifestations and subgroups.

	Subgroups															
	1 Anti-Ro52		2 Anti-U1RNP		3 Anti-PM/Scl		4 Anti-Mi2		5 Anti-Jo1		6 Anti-Jo1/Ro52		7 Anti-TIF1γ		8 None^a^	
	OR [95% CI]	p-value	OR [95% CI]	p-value	OR [95% CI]	p-value	OR [95% CI]	p-value	OR [95% CI]	p-value	OR [95% CI]	p-value	OR [95% CI]	p-value	OR [95% CI]	p-value
ILD	1.0 [0.4–3.4]	1.00	1.2 [0.4–5.3]	0.73	1.4 [0.4–9.1]	0.63	0.6 [0.2–2.1]	0.37	1.5 [0.4–9.6]	0.57	3.4 [0.7–62]	0.23	0.1 [0.1–0.4]	<0.001	1.5 [0.8–3.2]	0.24
Muscle weakness	0.7 [0.2–2.4]	0.49	0.6 [0.2–2.0]	0.32	0.6 [0.2–2.6]	0.43	0.5 [0.2–2.2]	0.29	0.7 [0.2–3.2]	0.63	3.5 [0.7–64]	0.23	0.7 [0.2–4.4]	0.61	2.4 [1.0–6.7]	0.08
Mechanic's hand	0.6 [0.3–1.2]	0.12	1.1 [0.5–2.5]	0.82	1.3 [0.5–3.9]	0.60	1.0 [0.4–3.4]	0.94	0.7 [0.3–1.5]	0.28	2.6 [1.0–8.2]	0.07	1.0 [0.3–4.5]	0.96	1.0 [0.6–1.7]	0.89
Heliotrope	1.3 [0.7–2.3]	0.39	0.9 [0.5–1.5]	0.62	2.2 [1.1–4.8]	0.03	12 [3.6–75]	<0.001	1.2 [0.7–2.1]	0.64	0.4 [0.2–0.7]	<0.001	22 [4.7–396]	0.003	0.5 [0.4–0.7]	<0.001
Gottron's	1.3 [0.7–2.3]	0.42	0.8 [0.5–1.4]	0.49	2.6 [1.3–5.9]	0.01	7.8 [2.8–33]	<0.001	1.3 [0.7–2.4]	0.40	0.4 [0.2–0.7]	<0.001	11 [3.1–66]	0.001	0.5 [0.4–0.7]	<0.001
Calcinosis	1.3 [0.6–2.6]	0.48	0.7 [0.3–1.3]	0.22	1.0 [0.4–2.1]	0.91	1.9 [0.7–4.1]	0.16	1.0 [0.5–1.9]	0.91	1.0 [0.5–2.2]	0.92	0.6 [0.2–1.8]	0.43	1.0 [0.7–1.6]	0.87
Raynaud	0.8 [0.4–1.7]	0.53	1.5 [0.7–3.8]	0.36	1.7 [0.6–6.0]	0.36	0.9 [0.3–3.1]	0.84	1.4 [0.6–3.8]	0.53	3.3 [1.2–11]	0.03	0.9 [0.3–3.9]	0.88	0.6 [0.4–1.0]	0.03
Arthritis	0.7 [0.3–1.7]	0.41	0.8 [0.4–2.1]	0.65	0.7 [0.3–2.3]	0.55	0.6 [0.2–2.1]	0.34	2.7 [0.8–17]	0.18	2.2 [0.7–9.3]	0.23	0.5 [0.2–1.9]	0.26	1.1 [0.7–2.0]	0.64
Dysphagia	0.9 [0.4–2.2]	0.87	0.7 [0.3–1.5]	0.28	0.8 [0.3–2.1]	0.55	0.9 [0.3–3.8]	0.82	1.3 [0.5–4.0]	0.62	0.7 [0.3–1.6]	0.35	1.3 [0.4–8.7]	0.69	1.4 [0.8–2.4]	0.19

Legend: ILD, interstitial lung disease.

All models are adjusted for sex, age at diagnosis as a quadratic term and recruiting center.

a None of the autoantibodies analysed for in this study.

### Genetic and clinical associations by serologically defined IIM subgroups

#### Subgroup 1: anti-Ro52-dominated

Subgroup 1 was among the most heterogeneous according to its serological profile, with the presence of 11 of the 14 autoantibodies analysed and dominated by anti-Ro52. DM, PM, OM, and IBM were the most frequent clinical subsets, and no significant difference in clinical manifestations were observed between subgroup 1 and the rest of the patients. Although initial significant genetic associations were observed with the *HLA-DRB1∗03* and *HLA-DRB1∗15* alleles, these disappeared after multiple correction testing (Supplementary Table S4).

#### Subgroup 2: anti-U1RNP-dominated

This subgroup, with 43% anti-U1RNP, was also heterogeneous concerning the serological profile, with the presence of 13 out of 14 autoantibodies analysed. DM, OM, PM, and anti-synthetase syndrome were the most frequent clinical subsets. No significant differences regarding clinical manifestations were observed when this group was compared with all others. *HLA-DRB1∗04, HLA-DRB1∗11, HLA-DRB1∗15, HLA-DQA1∗03,* and *HLA-DQB1∗03* alleles were overrepresented in this subgroup, while no signal was detected for class I amino acids after correction for multiple comparisons. The conditional analyses showed that the *HLA-DRB1∗15* association was independent of the *HLA-DRB1∗04, HLA-DRB1∗11, HLA-DQA1∗03,* and *HLA-DQB1∗03* signals (Supplementary Table S5).

#### Subgroup 3: anti-PM/Scl-dominated

This subgroup was dominated by patients positive for anti-PM/Scl, mostly DM, OM and PM patients. Significant associations were seen with class II alleles (*HLA-DRB1∗03, HLA-DQA1∗05,* and *HLA-DQB1∗02*) and correspondingly with amino acids in HLA class II but not in HLA class I molecules. These associations with HLA class II alleles were not independent of each other (Supplementary Table S6) and pointed toward the involvement of the ancestral haplotype 8.1 (see Supplementary Information 3). The most significant and strong amino acid associations in terms of OR were with alanine (A) at position −10 of HLA-DQB1 and lysine (K) at position 71 of HLA-DRB1 (Supplementary Table S5).

#### *Subgroup 4: anti-*Mi2*-dominated*

This subgroup, with all patients positive for anti-Mi2, was mostly comprised of patients with DM. *HLA-DRB1∗07* and *HLA-DQA1∗02* alleles were more common in this subgroup than in other subgroups. These associations were strong in terms of OR (95% CI) with 8.8 (5–15.7) for *HLA-DRB1∗07* and 7.0 (3.5–14) for *HLA-DQA1∗02*. Glutamine (Q) at position 74 of HLA-DRB1, encoded by the *HLA-DRB1∗07:01* allele*,* was positively associated with this subgroup and an association with HLA-A, with histidine (H) at position 74, was detected (Fig. 3, Supplementary Tables S4 and S5).

#### Subgroup 5: anti-Jo1-dominated

In this subgroup, all patients were positive for anti-Jo1, and they had the lowest median [IQR] age at diagnosis at 48 years [35–58] (Table 1). Clinically, most patients in this subgroup fulfilled the criteria for anti-synthetase syndrome. Nevertheless, no significant differences in clinical manifestations were observed between subgroup 5 and the rest of the patients. *HLA-DRB1∗03* was positively associated with this subgroup, as well as arginine (R) at position 74 of HLA-DRB1 and aspartic acid (D) at position 9 of HLA-B (Fig. 2, Fig. 3, Supplementary Tables S4 and S5).

#### Subgroup 6: anti-Jo1/Ro52-dominated

All the patients in this subgroup were positive for anti-Jo1 and anti-Ro52. Clinically, they all were classified as anti-synthetase syndrome with a significantly higher presence of Raynaud phenomenon compared to the other subgroups (Table 1, Table 2). *HLA-DRB1∗03, HLA-DQA1∗05, HLA-DQB1∗02* alleles, arginine (R) at position 74 of HLA-DRB1, and aspartic acid (D) at position 9 of HLA-B were positively associated with this subgroup (Fig. 2, Fig. 3, Supplementary Tables S4 and S5). As shown by conditional analyses, these associations were not independent of each other and corresponded to the ancestral haplotype 8.1 (Supplementary Information 3, Supplementary Table S6). Nevertheless, other positive associations with specific amino acids in the peptide-binding groove of HLA-B and HLA-C were observed, such as serine (S) or tryptophan (W) at position 97 of HLA-B (Fig. 3, Supplementary Table S5) suggesting an extended and complex genetic association pattern for this subgroup.

#### Subgroup 7: anti-TIF1γ-dominated

All patients in this subgroup were positive for anti-TIF1γ and were mainly classified clinically as DM. Positive associations were observed with *HLA* class II and alleles, including *HLA-DRB1∗07, HLA-DRB1∗01, HLA-DQA1∗02, HLA-DQB1∗05* alleles and with arginine (R) at position 71 of HLA-DRB1, threonine (T) at position 80 of HLA-B and phenylalanine (F) at position 9 of HLA-A (Fig. 2, Fig. 3, Supplementary Tables S4 and S5).

#### Subgroup 8: negative for analysed autoantibodies

Patients in this subgroup were negative for all analysed autoantibodies and had the highest median [IQR] age at diagnosis with 58 years [46–68] (Table 1). This subgroup had lower odds of developing DM rashes and Raynaud's phenomenon as compared with patients from all other subgroups. *HLA-DRB1∗13, HLA-DQA1∗01, HLA-DQB1∗06* alleles and presence of isoleucine (I) at position 95 and glutamic acid (E) at position 45 of HLA-B were positively associated with this subgroup (Fig. 2, Fig. 3, Supplementary Tables S4 and S5).

## Discussion

In this study, we aimed first to construct serologically defined subsets of IIM and then explore the associations of these subsets with genetic variations in the *HLA* region and clinical phenotypes, respectively. A general starting point of our analysis was that genetics, particularly *HLA*-based genetics, provide the most objective information on molecular mechanisms of pathogenic importance in different subsets of immune-mediated disease. Comparing subsets based on clinical and histopathological features to these serologically defined subgroups concerning different *HLA* variants might tell us which subgrouping method may best reflect the contribution of different pathogenic mechanisms. The results obtained using this analytic approach suggest that subgrouping IIM based on autoantibody profiles and *HLA* variants may reflect distinct pathogenetic mechanisms better than subgrouping based on traditional clinical/histopathological classifications (Fig. 1).

Our results provide a more comprehensive analysis of serologically defined subgrouping of IIM based on a large number of included patients, the autoantibody specificities analysed, and the methodological approach used than previously studied. Different autoantibody screening methods were allowed to maximize the cohort size available for serologic analysis, which may have resulted in over- or underrepresentation of certain autoantibodies depending on the techniques used.^20^ Moreover, even if autoantibody screening at diagnosis was preferred, screening at any point in the disease course was accepted, which may have reduced the detection of certain autoantibodies due to treatment or fluctuations with disease activity.^21^, ^22^, ^23^ We are also aware that the subgroup negative for all analysed autoantibodies may contain patients positive for new MSA, such as anti-FHL1, or autoantibodies that were either not analysed or removed from the analysis due to substantial missing data (e.g., anti-HMGCR, -Ku, -cN1A).^24^ Moreover, ethnic background was available for only 28% of the patients included, so ancestry could not be adjusted for in the different models. Despite the potential misclassification resulting from our autoantibody assessment strategy and possible presence of unmeasured confounders, this study allowed us to use unsupervised clustering to define IIM subgroups based on serological profiles, evaluate *HLA* associations, and contrast traditional clinical and histopathological classification.

Concerning genetic associations, most of the serologically defined subgroups showed associations with one or more *HLA* genetic variants. Notably, we observed several associations between the serologically defined subgroups and HLA class I amino acids. For example, the subgroup dominated by anti-Jo1/Ro52 showed signals from HLA-C and HLA-B amino acids, two of which were not found in the subgroup dominated by anti-Jo1 only, suggesting that besides the ancestral haplotype 8.1, other *HLA* haplotypes could be genetic contributors in subgroup 6. We observed that the *HLA-DRB1∗07* allele is associated with the subgroup dominated by anti-TIF1γ, observation that has not been reported before. Similarly, we found three signals from HLA-B amino acids and the association with *HLA-DRB1∗13, HLA-DRB1∗01* alleles in the subgroup negative for all analysed autoantibodies. These findings support the concept that unanalysed or unknown autoantibodies may be present in patients of subgroup 8.

Our study adds a different perspective by addressing autoantibody profiles instead of single specificities highlighting the importance of MAA in the presence or absence of MSA. Previous work has shown that *HLA* alleles are associated with several individual MSA/MAA in patients with IIM.^7^, ^8^, ^9^, ^10^ For example, in subgroup 2, dominated by anti-U1RNP, an association was seen with *HLA-DRB1∗15* and *HLA-DRB1∗11* alleles. This finding is in agreement with results from a Polish mixed-connective tissue disease cohort where the presence of anti-U1RNP antibodies associated with *HLA-DRB1∗15* allele (OR (95% CI) 6.1 (4.6–8.1)).^25^ Moreover, subgroups dominated by anti-Ro52 (i.e., subgroups 1 and 6) showed an association with the *HLA-DRB1∗03* allele in our study*,* similar to associations found in specific subgroups dominated by anit-Ro60/52 in Sjögren syndrome^26^ and systemic lupus erythematosus.^27^ Other genetic variants in the *HLA* loci may be associated with the subgroups defined by autoantibodies. For example, low copy numbers of complement C4 and low C4 protein levels were related to the presence of anti-Ro/La, anti-Jo1, and anti-PM/Scl in IIM.^28^^,^^29^ Since these C4 associations are not completely independent from the association with *HLA-DRB1∗03*, both types of genetic variations may contribute independently and interactively to the autoantibody-defined IIM subgroups.

Our results reinforce the idea that rheumatic diseases share similar genetic risk factors (i.e., *HLA*) which predispose to certain autoantibody specificities and that there is a reciprocal relationship between specific genetic *HLA* variations and the presence of particular autoantibodies.^26^, ^27^, ^28^, ^29^

Although a significant number of patients were included in this study, we might still lack statistical power to detect some significant associations. As a result, large OR estimates and CI limits for some of the smaller subgroups could be explained by a sparse data bias (i.e., lack of adequate case numbers for some combination of risk factor and outcome levels).^30^ In addition, we chose to use two-digit *HLA* allele resolution and screen imputed amino acids to reduce the multiple correction burden. Still, four-digit *HLA* resolution might reveal differences not seen in using two-digit resolution. Such discrepancies have previously been shown in studies comparing adult and juvenile-onset IIM patients.^10^

**Fig. 4:**
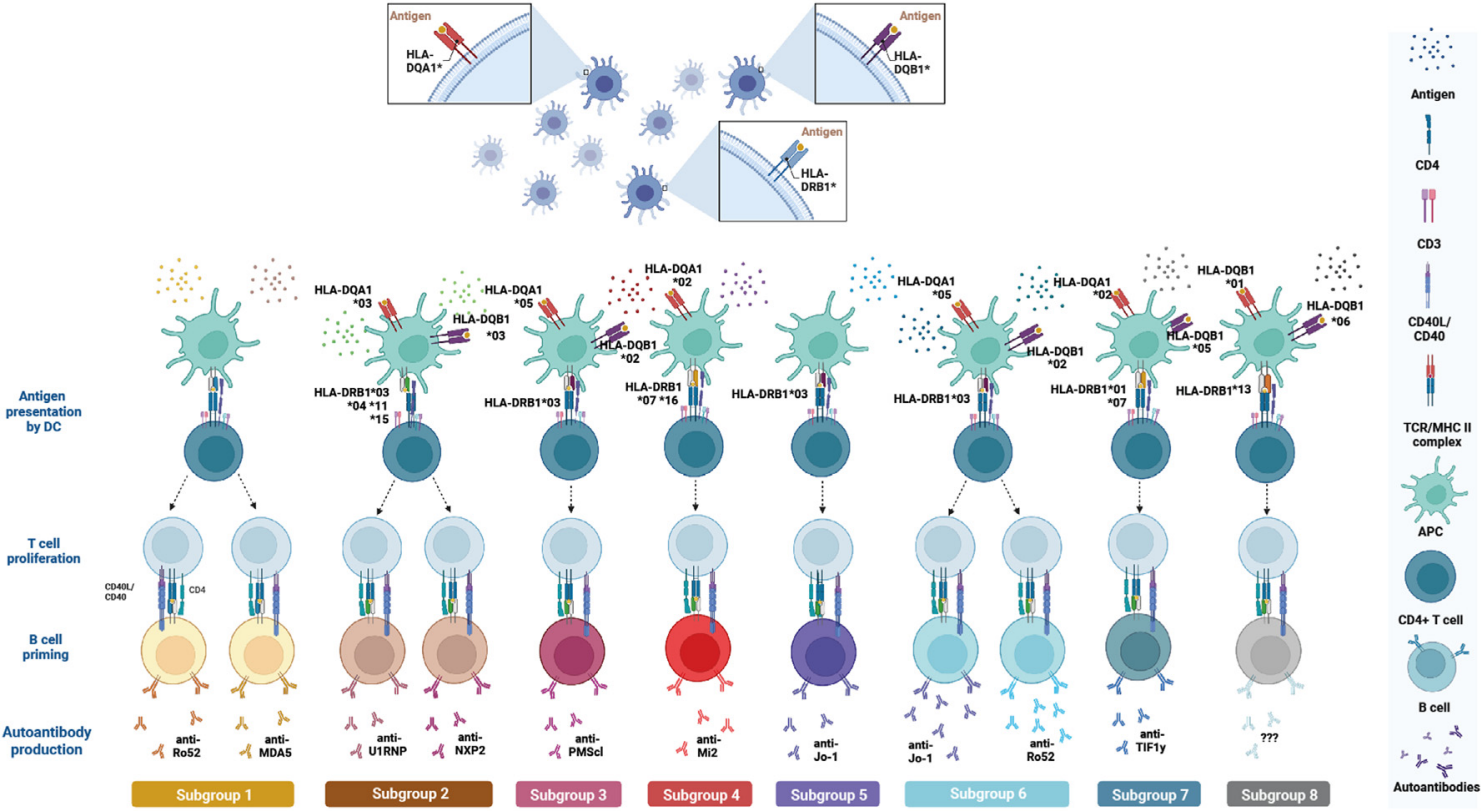
**Representation of suggested mechanisms for autoantibody production in IIM**. Different HLA molecules, determined by their genetic variability, influence antigen presentation, T-cell differentiation and proliferation, and B-cells priming, leading to specific autoantibody production in IIM. Based on the expression of particular *HLA* alleles, distinct autoantibodies are produced. In some cases, this differential expression of MHCII would lead to B-cell priming and production of both myositis-specific autoantibodies (MSA) (ex: anti-Jo1 or anti-MDA5) and myositis-associated autoantibodies (MAA) (ex: anti-Ro52) in the same individual (subgroup 1 or 6). Created with BioRender.com

Understanding what triggers the HLA-linked production of autoantibodies, the break of self-tolerance in autoimmune diseases, and how these may contribute to disease development and phenotypes is challenging. Our results suggest that distinct pathophysiological pathways may be underlying the production of several autoantibodies in the same individual (e.g., anti-Jo1 and anti-Ro52). The mechanisms behind the co-occurrence of autoantibodies in the IIM subgroups described herein may result from the exposure of several cryptic antigens after cell apoptosis in inflamed and damaged tissue, such as muscle, skin, or lung, leading to a concerted loss of peripheral tolerance and presence of autoreactive T-cells^31^, ^32^, ^33^, ^34^–for an illustration of the concept, see Fig. 4.

Our findings indicate that we need to systematically include MAAs in autoantibody profiling in the clinic. The fact that worst outcomes were previously described in anti-Jo1 patients with anti-Ro52 while we report differential genetic associations for *HLA* class I between isolated anti-Jo1 and anti-Jo1/Ro52 is an argument for broader systematic autoantibody screening.^5^ The combination of antibody profiles with clinical features improves clinical phenotyping. This is clearly depicted in our study by the lack of differentiating clinical features between our autoantibody-defined subgroups, which is not surprising as IIM individuals often share similar clinical manifestations (e.g., ILD). Moreover, our data did not allow for severity or longitudinal assessment of those clinical features that might have helped identify differences between these subgroups regarding clinical outcomes. Some findings in muscle tissue of clinically defined subgroups are not distinct and can overlap while some histopathological features are strongly associated with certain MSA (e.g., anti-Jo1, -TIF1y).^35^^,^^36^ In that context, it could have been interesting to explore associations between muscle histopathology features and autoantibody-defined subgroups, however no histopathological data was collected in this study to allow such analysis. Nonetheless, results from our study suggest that future classification of IIM should include serological profiles, which could contribute to earlier diagnosis, as evidenced by older age at diagnosis of autoantibody negative cases, as well as better reflect pathogenetic mechanisms than the traditional subdivisions based mainly on clinical and histopathological features, although this needs to be further studied. Disease classification is a fundamental component of clinical and translational research and can be the key to discovering targeted therapies that could improve the outcomes for IIM patients. Thus, it is important that more autoantibodies are included in future classification criteria as they are linked to distinct genetic susceptibility.

### Conclusion

Autoantibody-defined subgroups of IIM have more robust and consistent *HLA* class II and I associations than subgroups based mainly on clinico-pathological features. In this large IIM cohort, associations between autoantibody-defined subgroups considering multiple specificities and *HLA* class II and class I were described. Our results support systematic screening of MAA/MSAs in the clinic and an autoantibody-based IIM classification that would contribute to understanding risk factors and pathogenic mechanisms in IIM.

## Contributors

VL, ASGF and LMDG designed the study. SR, OK, SSZ, HM, LPD, HA, MK, ST, NM, JL, JV, HC, MH, LP, and IEL contributed to sample collection, clinical assessment, genotyping, and autoantibody measurements. LR, KLT, ACS, IEL, and MWH are principal investigators of The Dissect Consortium and The Immunoarray Development Consortium, contributing to sample collection and genotyping. JKS contributed by providing access to data and coordinating the collaboration with The Dissect Consortium and The Immunoarray Development Consortium. VL gathered, curated, and quality-controlled the data. VL, MB, LP, and LMDG performed analyses. VL, ASGF, and LMDG interpreted the results, prepared the tables and figures, and drafted the first version of the manuscript. VL and LMDG have accessed and verified the underlying data. All the authors read, edited, and approved the study.

## Data sharing statement

Given the sensitive nature of the data, it is necessary to request access based on approval and agreements with MYONET registry and the representatives from each center. For more information, contact the corresponding authors.

## Declaration of interests

Professor Lundberg has received consulting fees from Corbus Pharmaceuticals, Inc and research grants from Astra Zeneca and has been serving on the advisory board for Astra Zeneca, Bristol Myers Squibb, EMD Serono Research & Development Institute, Argenx, Octapharma, Kezaar, Orphazyme, Pfizer and Janssen and has stock shares in Roche and Novartis. Professor Vencovský has received speaker fees from Abbvie, Biogen, Boehringer, Eli Lilly, Gilead, MSD, Novartis, Pfizer, Roche, Sanofi, UCB, Werfen; and has received consulting fees from Abbvie, Argenx, Boehringer, Eli Lilly, Gilead, Octapharma, Pfizer, UCB. Professor Chinoy has received personal compensation for activities with Novartis, UCB, Eli Lilly, Biogen, Orphazyme, Astra Zeneca, Pfizer, Kezar Life Sciences, Galapagos, Argenx, GSK as a speaker, advisory board member or consultancy; grants via The University of Manchester from Eli Lilly and UCB; travel support from Abbvie and Janssen. Dr Mann has received honoraria from Abbvie, Biogen, BMS, Eli Lilly, MSD, Janssen, Novartis, UCB, Pfizer, SOBI and travel support by Abbvie. Dr Tansley received payment from Boehringer Ingelheim as a speaker. Dr Rönnblom received support from The Swedish Research Council (2018–02399), Reumatikerförbundet (R-939716) and GV:80 (FAI-2020-0658) foundation. Dr Holmqvist received support from The Swedish Research Council, Stockholm Regional Council (ALF), Reumatikerförbundet, Konung Gustaf V:80 year foundation, The Swedish Cancer Association and was a member of Medical Advisory Board of The Myositis Association. Dr Lamb received support from the Medical Research Council UK and Myositis UK. Other authors declare no competing interests.

## Appendix A Supplementary data

EBIOM-D-23-00918 Consortia.docx
