# Supplementary material for: Distinct HLA associations with autoantibody-defined subgroups in idiopathic inflammatory myopathies
Source: eBioMedicine. 2023 Sep 26;96:104804. doi: 10.1016/j.ebiom.2023.104804 (PMC10550566; doi:10.1016/j.ebiom.2023.104804)
Supplement: EBIOM-D-23-00918 Consortia.docx [file mmc1.docx]

**Members of the DISSECT Consortium**

| **First name** | **Surnames** |
| --- | --- |
| Matteo | Bianchi |
| Sergey V | Kozyrev |
| Johanna K | Sandling |
| Lars | Rönnblom |
| Maija-Leena | Eloranta |
| Ann-Christine | Syvänen |
| Dag | Leonard |
| Johanna | Dahlqvist |
| Maria | Lidén |
| Argyri | Mathioudaki |
| Jennifer RS | Meadows |
| Jessika | Nordin |
| Gunnel | Nordmark |
| Ingrid E | Lundberg |
| Antonella | Notarnicola |
| Leonid | Padyukov |
| Anna | Tjärnlund |
| Maryam | Dastmalchi |
| Daniel | Eriksson |
| Øyvind | Molberg |
| Helena | Andersson |
| Kerstin | Lindblad-Toh |
| Fabiana HG | Farias |
| Marie | Wahren-Herlenius |
| Awat | Jalal |
| Balsam | Hanna |
| Helena | Hellström |
| Tomas | Husmark |
| Åsa | Häggström |
| Anna | Svärd |
| Thomas | Skogh |
| Louise | Pyndt Diederichsen |
| Janine A | Lamb |
| Simon | Rothwell |
| Hector | Chinoy |
| Robert G | Cooper |

**Members of the ImmunoArray Development Consortium**

| **First name** | **Surnames** |
| --- | --- |
| Kerstin | Lindblad-Toh |
| Gerli | Rosengren Pielberg |
| Anna | Lobell |
| Åsa | Karlsson |
| Eva | Murén |
| Kerstin M | Ahlgren |
| Lars | Rönnblom |
| Maija-Leena | Eloranta |
| Göran | Andersson |
| Nils | Landegren |
| Olle | Kämpe |
| Peter | Söderkvis |
