# Supplementary material for: Distinct HLA associations with autoantibody-defined subgroups in idiopathic inflammatory myopathies
Source: eBioMedicine. 2023 Sep 26;96:104804. doi: 10.1016/j.ebiom.2023.104804 (PMC10550566; doi:10.1016/j.ebiom.2023.104804)
Supplement: Supplementary Material [file mmc2.docx]

**SUPPLEMENTARY INFORMATION AND SUPPLEMENTARY TABLES**

**Distinct *HLA* associations with autoantibody-defined subgroups in idiopathic inflammatory myopathies**

**Supplementary Information 1.** The Dissect Consortium and the Immunoarray Development Consortium Collaborators.

**Supplementary Information 2.** Targeted DNA resequencing and analysis of the DISSECT data.

**Supplementary Information 3.** Software and packages used for data analyses.

**Supplementary Figure 1.** tSNE and Silhouette plots representing the subgroups selected.

**Supplementary Table 1.** MYONET registry definitions.

**Supplementary Table 2.** Method for autoantibody testing and *HLA* alleles determination by recruiting centers.

**Supplementary Table 3.** Cohort characteristics stratified by centers.

**Supplementary Table 4.** Significant *HLA-DQA1*, *HLA*-*DQB1* and *HLA*-*DRB1* alleles associations from the meta-analyses.

**Supplementary Table 5.** Significant amino acids associations from the meta-analyses clumped by linkage disequilibrium.

**Supplementary Table 6.** Conditioned analyses for class II alleles, including UK, Scandinavia, and Czech Republic populations.

**Supplementary Information 1.** The Dissect Consortium and The Immunoarray Development Consortium Collaborators^1-3^.

**Members of the DISSECT Consortium**

| **First names** | **Surnames** |
| --- | --- |
| Matteo | Bianchi |
| Sergey V | Kozyrev |
| Johanna K | Sandling |
| Lars | Rönnblom |
| Maija-Leena | Eloranta |
| Ann-Christine | Syvänen |
| Dag | Leonard |
| Johanna | Dahlqvist |
| Maria | Lidén |
| Argyri | Mathioudaki |
| Jennifer RS | Meadows |
| Jessika | Nordin |
| Gunnel | Nordmark |
| Ingrid E | Lundberg |
| Antonella | Notarnicola |
| Leonid | Padyukov |
| Anna | Tjärnlund |
| Maryam | Dastmalchi |
| Daniel | Eriksson |
| Øyvind | Molberg |
| Helena | Andersson |
| Kerstin | Lindblad-Toh |
| Fabiana HG | Farias |
| Marie | Wahren-Herlenius |
| Awat | Jalal |
| Balsam | Hanna |
| Helena | Hellström |
| Tomas | Husmark |
| Åsa | Häggström |
| Anna | Svärd |
| Thomas | Skogh |
| Louise | Pyndt Diederichsen |
| Janine A | Lamb |
| Simon | Rothwell |
| Hector | Chinoy |
| Robert G | Cooper |

**Members of the ImmunoArray Development Consortium**

| **First names** | **Surnames** |
| --- | --- |
| Kerstin | Lindblad-Toh |
| Gerli | Rosengren Pielberg |
| Anna | Lobell |
| Åsa | Karlsson |
| Eva | Murén |
| Kerstin M | Ahlgren |
| Lars | Rönnblom |
| Maija-Leena | Eloranta |
| Göran | Andersson |
| Nils | Landegren |
| Olle | Kämpe |
| Peter | Söderkvist |

**Supplementary Information 2.** Targeted DNA resequencing and analysis of the DISSECT data.

DNA extracted from blood samples of Swedish, Danish, Norwegian, and UK patients underwent targeted resequencing as described previously^3^. Briefly, sequence capture was performed with a custom NimbleGen array and paired-end short reads Illumina next-generation sequencing with an HiSeq 2500. The array included 1853 genes involved in immune function, inflammation, and autoimmunity, for which both coding and potentially regulatory regions were targeted. The design and the implementation of the array have been fully outlined in a previous study.^4^

Raw resequencing data were mapped using the mem algorithm of the Burrows-Wheeler Alignment (v.0.7.12)^5^ to the human genome build hg19/GRCh37. GATK v3.3.0 best practices^6-8^ were applied before variant calling, which was performed with HaplotypeCaller in gVCF mode in the two cohort datasets separately (i.e., Scandinavia and UK). GenotypeGVCFs ws then used to perform joint genotyping in the two datasets, and only biallelic single nucleotide variants (SNVs) were considered in further analyses. Subsequently, GATK 3.3.0 VariantRecalibrator was applied with genotypes filtered based on probability scores, read depth and genotype quality. Further quality control was implemented, based on individual ancestry prediction, relatedness, resequencing-related parameters (e.g., missingness and heterozygosity levels, transitions/transversions ratio, singleton counts) and variant-based quality parameters (e.g., allelic balance, Hardy-Weinberg equilibrium, missingness level) to retain only individuals and SNVs of high quality.

Finally, the genotypes of all SNVs located in the extended MHC region (chr6:27339429-34586722) were extracted from the individual samples and subjected to classical *HLA* alleles and amino acids imputation using the software SNP2HLA^9^ and the T1DGC reference panel.^9^

**Supplementary Information 3.** Software and packages used for data analyses.

The genetic association analyses were performed using PLINK (version 1·9).^10^ All other statistical analyses were performed using R versions 3·6·1 and 4·1·1^11^, notably the *cluster,*^12^ *MASS^13^* and *meta*^14^ packages.

Confidence intervals calculated from the *MASS* and *meta* packages were obtained using the profile likelihood confidence intervals and standard normal method, respectively.

**Supplementary Figure 1.** tSNE and Silhouette plots representing the subgroups selected.

**Legend**: (**a**) tSNE representation of subgroups based on autoantibody positivity**.** One dot represents one patient. The colour intensity is due to points overlap. Individuals with no positivity for included autoantibodies in this study were considered as a separate subgroup, the 8^th^ subgroup, not included in this panel. (**b**) Plot of the silhouette width used to decide on the optimal number of subgroups. Silhouette width values closer to one indicates the optimal quantity of subgroups for the given data set. Based on this, an average silhouette width of 0.67 corresponding to 7 clusters (k) was chosen. (**c**) Silhouette plot showing consistency of data within each of the seven subgroups.

PAM, partition around medoid

**Supplementary Table 1.** MYONET registry definitions^15^.

| **Features** | **Definitions** |
| --- | --- |
| **Interstitial lung disease** | Radiologic (chest x-ray or chest CT scan) documentation of inflammation or scarring (fibrosis) of the parenchyma of the lung, **and** abnormal PFT attributable to inflammatory process or pulmonary fibrosis. |
| **Mechanic’s hands** | Scaling or cracking of the skin over the lateral or palmar aspects of the fingers or thumbs |
| **Gottron’s papules/plaques** | Papules: Erythematous to violaceous papules over the extensor surfaces of joints, which are sometimes scaly. May occur over the finger joints, elbows, knees, malleoli and toes.  Signs: Erythematous to violaceous macules over the extensor surfaces of joints, which are not palpable |
| **Heliotrope rash** | Purple, lilac-coloured or erythematous patches over the eyelids or in a periorbital distribution, often associated with periorbital oedema |
| **Ulceration** | Extensive injury to dermis or deeper due to dermatomyositis |
| **Calcinosis** | Dystrophic calcium deposits, observed clinically or by imaging, which involves the skin, subcutaneous tissue, fascia or muscle |
| **Raynaud** | Discoloration of fingertips or other acral areas (two or three colours) to emotion or cold |
| **Arthritis** | Inflammation, including swelling, warmth, tenderness, and/or redness of one or more joints detected by physical exam |
| **Dysphagia** | Difficulty in swallowing or objective evidence of abnormal motility of the oesophagus |

**Supplementary Table 2.** Methods for autoantibody testing and *HLA* alleles determination by recruiting centers.

|  | **UK Myositis Network (UKMYONET)** | **Institute of Rheumatology** | **Karolinska University Hospital** | **Odense & Copenhagen University Hospitals** | **Oslo University Hospital** |
| --- | --- | --- | --- | --- | --- |
| **Location** | UK | Czech Republic | Sweden | Denmark | Norway |
| **Autoantibodies** | | | | | |
| **IP*** | Anti-Jo1, P-L12, -PL7, -OJ, -EJ, -Mi2, -NXP2, -MDA5, - TIF1$\gamma$ -SAE, -SRP, -PM/Scl, and -U1RNP/Sm | Anti-Jo1, P-L12, -PL7, -OJ, -EJ, -Mi2, -NXP2, -MDA5, -TIF1$\gamma$,-SAE, -SRP, -PM/Scl, and -U1RNP/Sm | Anti-Jo1, P-L12, -PL7, -OJ, -EJ, -Mi2, -NXP2, -MDA5, -TIF1$\gamma$,-SAE, -SRP, -PM/Scl, and -U1RNP/Sm | None | None |
| **Lineblot kit** | None | Euroline Myositis Profile 3, Euroimmun, Lübeck, Germany | Euroline Myositis Profile 3 and 4, Euroimmun, Lübeck, Germany | Euroline Myositis Profile 4, Euroimmun, Lübeck, Germany | Euroline Myositis Profile 4, Euroimmun, Lübeck, Germany |
| **ELISA** | Ro52 Orgentec Diagnostika GmbH, Mainz, Germany | None | None | EliA Ro Well 14-5503-01, Phadia, ThermoScientific | None |
| ***HLA* alleles determination** | | | | | |
| **Genotyping** | Illumina immunochip array^16†^ | Luminex multiplex assay^17^ | Sequence-specific primer polymerase chain reaction assay (DR low-resolution kit; Olerup SSP)^18^ |  |  |
| **Imputation**** | SNP2HLA | SNP2HLA | SNP2HLA | SNP2HLA | SNP2HLA |

**Legend:**

*Samples with a 140kDa band were further assessed by ELISA as anti-NXP2 and anti-MDA5 migrate at the same molecular weight.^19^

******Using the single nucleotide variants extracted from the Dissect Consortium targeted sequencing data.

^†^Performed in the Centre for Genetics and Genomics Versus Arthritis, University of Manchester, UK

Lineblot results were reported positive if >11 or +.

IP, immunoprecipitation; ELISA, enzyme-linked immunosorbent assay

**Supplementary Table 3.** Cohort characteristics stratified by centers.

|  | **Centers** | | | | |  |
| --- | --- | --- | --- | --- | --- | --- |
|  | **United Kingdom** | **Sweden** | **Czech Republic** | **Denmark** | **Norway** | **All** |
| **n** | 724 (54) | 296 (22) | 210 (16) | 83 (6) | 35 (3) | 1348 (100) |
| **Female** (%) | 472 (65) | 187 (63) | 149 (71) | 53 (64) | 21 (60) | 882 (65) |
| **Age at diagnosis,** median [IQR] | 57 [44-60] | 56 [44-69] | 53 [41-61] | 54 [44-59] | 48 [40-57] | 55 [42-65] |
| **IIM subsets** |  |  |  |  |  |  |
| DM | 188 (26) | 80 (27) | 88 (42) | 17 (21) | 10 (29) | 383 (28) |
| PM | 120 (17) | 61 (21) | 47 (22) | 35 (42) | 6 (17) | 269 (20) |
| Anti-synthetase | 156 (22) | 62 (21) | 60 (29) | 28 (34) | 11 (31) | 317 (24) |
| Overlap myositis | 118 (16) | 35 (12) | 9 (4) | 1 (1) | 0 | 163 (12) |
| IBM | 104 (14) | 41 (14) | 1 (1) | 0 | 0 | 146 (11) |
| IMNM | 31 (4) | 7 (2) | 5 (2) | 1 (1) | 6 (17) | 50 (4) |
| Juvenile onset DM | 7 (1) | 7 (2) | 0 | 1 (1) | 1 (3) | 16 (1) |
| Unspecific | 0 | 3 (1) | 0 | 0 | 1 (3) | 4 (0) |
| **Autoantibodies** |  |  |  |  |  |  |
| Anti-Jo1 | 125 (17) | 57 (19) | 56 (27) | 21 (25) | 7 (20) | 266 (20) |
| Anti-PL7 | 9 (1) | 4 (1) | 3 (1) | 1 (1) | 3 (9) | 20 (1·5) |
| Anti-PL12 | 5 (0·7) | 2 (0·7) | 0 | 1 (1) | 2 (6) | 10 (0·7) |
| Anti-EJ | 0 | 1 (0·3) | 0 | 1 (1) | 0 | 2 (0·1) |
| Anti-OJ | 6 (0·8) | 1 (0·3) | 0 | 0 | 0 | 7 (0·5) |
| Anti-TIF1$\gamma$ | 39 (5) | 30 (10) | 21 (10) | 1 (1) | 1 (3) | 92 (7) |
| Anti-Mi2 | 40 (6) | 7 (2) | 17 (8) | 1 (1) | 5 (14) | 70 (5) |
| Anti-SAE1 | 18 (3) | 10 (3) | 2 (1) | 0 | 1 (3) | 31 (2) |
| Anti-NXP2 | 14 (2) | 8 (3) | 1 (0·5) | 2 (2) | 0 | 25 (2) |
| Anti-MDA5 | 6 (0·8) | 8 (3) | 6 (3) | 1 (1) | 1 (3) | 22 (2) |
| Anti-SRP | 15 (2) | 9 (3) | 7 (3) | 5 (6) | 4 (11) | 40 (3) |
| Anti-Ro52 | 124 (17) | 65 (22) | 64 (31) | 28 (34) | 12 (34) | 293 (22) |
| Anti-PM/Scl | 62 (9) | 27 (9) | 21 (10) | 6 (7) | 3 (9) | 119 (9) |
| Anti-U1RNP | 53 (7) | 21 (7) | 7 (3) | 0 | 1 (3) | 82 (6) |
| None^†^ | 302 (58) | 107 (36) | 62 (30) | 39 (47) | 9 (26) | 519 (39) |
| **Legend**: IIM, idiopathic inflammatory myopathy; DM, dermatomyositis; PM, polymyositis; IBM, inclusion body myositis; IMNM, Immune-mediated necrotising myositis.  ^†^None of the autoantibodies screened for in this study. | | | | | | |

| **Supplementary Table 4.** *HLA-DQA1, HLA-DQB1,* and *HLA-DRB1* alleles associations with subgroups of IIM patients. | | | | | | |
| --- | --- | --- | --- | --- | --- | --- |
| **Alleles** | **Population** | **OR [95% CI]** | **P-value** | **FDR** | **Q** | **I^2^** |
| **Subgroup 1 (anti-Ro52)** | | | | | | |
| *HLA-DRB1*03* | Scandinavia | 1·45 [0·82-2·49] | 0·21 |  |  |  |
|  | UK | 1·60 [0·89-2·88] | 0·12 |  |  |  |
|  | Czech Republic | 1·84 [0·87-3·90] | 0·11 |  |  |  |
|  | Meta-analysis | 1·57 [1·10-2·25] | 0·01 | 0·06 | 0·86 | 0 |
| *HLA-DRB1*15* | Scandinavia | 1·66 [0·88-3·12] | 0·12 |  |  |  |
|  | UK | 1·89 [0·94-3·78] | 0·07 |  |  |  |
|  | Czech Republic | 1·52 [0·57-4·11] | 0·40 |  |  |  |
|  | Meta-analysis | 1·71 [1·12-2·62] | 0·01 | 0·06 | 0·93 | 0 |
| **Subgroup 2 (anti-U1RNP​)** | | | | | | |
| *HLA-DQA1*03* | Scandinavia | 1·45 [0·80-2·65] | 0·22 |  |  |  |
|  | UK | 1·82 [0·97-3·41] | 0·06 |  |  |  |
|  | Czech Republic | 6·43 [0·80-51·61] | 0·08 |  |  |  |
|  | Meta-analysis | 1·72 [1·12-2·62] | 0·01 | 0·04 | 0·39 | 0 |
| *HLA-DQB1*02* | Scandinavia | 0·73 [0·39-1·35] | 0·31 |  |  |  |
|  | UK | 0·35 [0·18-0·67] | 2·0$\times$10^-3^ |  |  |  |
|  | Czech Republic | 0·54 [0·10-3·00] | 0·48 |  |  |  |
|  | Meta-analysis | 0·52 [0·35-0·80] | 3·0$\times$10^-3^ | 0·02 | 0·28 | 21·8 |
| *HLA-DQB1*03* | Scandinavia | 1·45 [0·87-2·42] | 0·16 |  |  |  |
|  | UK | 1·96 [1·09-3·51] | 0·03 |  |  |  |
|  | Czech Republic | 4·36 [1·02-18·62] | 0·05 |  |  |  |
|  | Meta-analysis | 1·76 [1·21-2·55] | 3·0$\times$10^-3^ | 0·02 | 0·33 | 8·9 |
| *HLA-DRB1*03* | Scandinavia | 0·77 [0·45-1·32] | 0·34 |  |  |  |
|  | UK | 0·36 [0·21-0·61] | 1·7$\times$10^-4^ |  |  |  |
|  | Czech Republic | 0·27 [0·06-1·26] | 0·10 |  |  |  |
|  | Meta-analysis | 0·50 [0·35-0·72] | 2·7$\times$10^-4^ | 2·0$\times$10^-3^ | 0·10 | 56·4 |
| *HLA-DRB1*04* | Scandinavia | 1·23 [0·71-2·13] | 0·47 |  |  |  |
|  | UK | 1·56 [0·99-2·45] | 0·06 |  |  |  |
|  | Czech Republic | 3·76 [1·02-13·9] | 0·05 |  |  |  |
|  | Meta-analysis | 1·51 [1·10-2·11] | 0·02 | 0·05 | 0·30 | 17·6 |
| *HLA-DRB1*11* | Scandinavia | 1·17 [0·54-2·57] | 0·69 |  |  |  |
|  | UK | 2·00 [1·04-3·88] | 0·04 |  |  |  |
|  | Czech Republic | 3·96 [1·22-12·86] | 0·02 |  |  |  |
|  | Meta-analysis | 1·85 [1·16-2·94] | 0·01 | 0·04 | 0·23 | 32·4 |
| *HLA-DRB1*15* | Scandinavia | 1·54 [0·84-2·83] | 0·17 |  |  |  |
|  | UK | 2·81 [1·64-4·79] | 1·6$\times$10^-4^ |  |  |  |
|  | Czech Republic | 1·10 [0·24-5·00] | 0·90 |  |  |  |
|  | Meta-analysis | 2·07 [1·40-3·05] | 2·5$\times$10^-4^ | 2·0$\times$10^-3^ | 0·24 | 29 |
| **Subgroup 3 (anti-PM/Scl)** | | | | | | |
| *HLA-DQA1*05* | Scandinavia | 2·08 [0·88-4·89] | 0·09 |  |  |  |
|  | UK | 2·67 [1·34-5·31] | 0·01 |  |  |  |
|  | Czech Republic | 2·05 [0·59-7·13] | 0·26 |  |  |  |
|  | Meta-analysis | 2·36 [1·44-3·85] | 6·0$\times$10^-4^ | 3·0$\times$10^-3^ | 0·88 | 0 |
| *HLA-DQB1*02* | Scandinavia | 4·16 [1·59-10·88] | 4·0$\times$10^-3^ |  |  |  |
|  | UK | 5·12 [2·20-11·93] | 2·0$\times$10^-4^ |  |  |  |
|  | Czech Republic | 9·85 [2·18-44·61] | 3·0$\times$10^-3^ |  |  |  |
|  | Meta-analysis | 5·23 [2·91-9·39] | 3·0$\times$10^-8^ | 3·0$\times$10^-7^ | 0·64 | 0 |
| *HLA-DRB1*03* | Scandinavia | 2·81 [1·27-6·19] | 0·01 |  |  |  |
|  | UK | 3·16 [1·71-5·84] | 2·0$\times$10^-4^ |  |  |  |
|  | Czech Republic | 5·27 [2·07-13·41] | 5·0$\times$10^-4^ |  |  |  |
|  | Meta-analysis | 3·40 [2·21-5·23] | 2·4$\times$10^-8^ | 1·7$\times$10^-7^ | 0·57 | 0 |
| **Subgroup 4 (anti-Mi2)** | | | | | | |
| *HLA-DQA1*02* | Scandinavia | 5·87 [1·32-26·15] | 0·02 |  |  |  |
|  | UK | 5·82 [2·42-13·97] | 1·0$\times$10^-4^ |  |  |  |
|  | Czech Republic | 17·20 [3·26-90·74] | 8·0$\times$10^-4^ |  |  |  |
|  | Meta-analysis | 7·02 [3·53-13·96] | 2·9$\times$10^-8^ | 2·3$\times$10^-7^ | 0·51 | 0 |
| *HLA-DQA1*05* | Scandinavia | 0·08 [0·01-0·72] | 0·02 |  |  |  |
|  | UK | 0·23 [0·09-0·60] | 3·0$\times$10^-3^ |  |  |  |
|  | Czech Republic | 0·15 [0·04-0·57] | 6·0$\times$10^-3^ |  |  |  |
|  | Meta-analysis | 0·18 [0·087-0·38] | 4·8$\times$10^-6^ | 1·9$\times$10^-5^ | 0·65 | 0 |
| *HLA-DRB1*03* | Scandinavia | 0·23 [0·05-1·09] | 0·06 |  |  |  |
|  | UK | 0·12 [0·04-0·35] | 1·0$\times$10^-4^ |  |  |  |
|  | Czech Republic | 0·07 [0·01-0·55] | 0·01 |  |  |  |
|  | Meta-analysis | 0·13 [0·06-0·3] | 9·1$\times$10^-7^ | 4·5$\times$10^-6^ | 0·66 | 0 |
| *HLA-DRB1*07* | Scandinavia | 7·48 [1·89-29·66] | 4·0$\times$10^-3^ |  |  |  |
|  | UK | 7·67 [3·73-15·76] | 3.0$\times$10^-8^ |  |  |  |
|  | Czech Republic | 17·09 [4·38-66·69] | 4·4$\times$10^-5^ |  |  |  |
|  | Meta-analysis | 8·82 [4·95-15·72] | 1·6$\times$10^-13^ | 1·6$\times$10^-12^ | 0·58 | 0 |
| *HLA-DRB1*16* | Scandinavia | 6·02 [0·64-56·26] | 0·12 |  |  |  |
|  | UK | 1·91 [0·23-15·88] | 0·55 |  |  |  |
|  | Czech Republic | 3·50 [0·97-12·71] | 0·06 |  |  |  |
|  | Meta-analysis | 3·41 [1·27-9·16] | 0·02 | 0·05 | 0·76 | 0 |
| **Subgroup 5 (anti-Jo1)** | | | | | | |
| *HLA-DRB1*03* | Scandinavia | 2·38 [1·15-4·90] | 0·02 |  |  |  |
|  | UK | 2·01 [1·17-3·47] | 0·01 |  |  |  |
|  | Czech Republic | 2·09 [0·87-5·01] | 0·10 |  |  |  |
|  | Meta-analysis | 2·13 [1·44-3·14] | 1·0$\times$10^-4^ | 1·0$\times$10^-3^ | 0·94 | 0 |
| **Subgroup 6 (anti-Jo1/-Ro52)** | | | | | | |
| *HLA-DQA1*05* | Scandinavia | 1·68 [0·93-3·04] | 0·09 |  |  |  |
|  | UK | 3·04 [1·46-6·35] | 3·0$\times$10^-3^ |  |  |  |
|  | Czech Republic | 1·67 [0·75-3·71] | 0·21 |  |  |  |
|  | Meta-analysis | 2·00 [1·34-2·98] | 7·0$\times$10^-4^ | 5·0$\times$10^-3^ | 0·41 | 0 |
| *HLA-DQB1*02* | Scandinavia | 2·32 [1·21-4·45] | 0·01 |  |  |  |
|  | UK | 3·11 [1·38-7·03] | 6·0$\times$10^-3^ |  |  |  |
|  | Czech Republic | 1·12 [1·49-2·57] | 0·79 |  |  |  |
|  | Meta-analysis | 2·07 [1·34-3·19] | 1·0$\times$10^-3^ | 5·0$\times$10^-3^ | 0·20 | 37·3 |
| *HLA-DRB1*03* | Scandinavia | 1·86 [1·03-3·37] | 0·04 |  |  |  |
|  | UK | 5·31 [2·51-11·21] | 1·0$\times$10^-5^ |  |  |  |
|  | Czech Republic | 1·59 [0·83-3·06] | 0·16 |  |  |  |
|  | Meta-analysis | 2·44 [1·2-4·97] | 0·01 | 1·4$\times$10^-4^ | 0·04 | 69·3 |
| **Subgroup 7 (anti-TIF1**$\boldsymbol{\gamma}$**)** | | | | | | |
| *HLA-DQA1*02* | Scandinavia | 5·23 [1·88-14·55] | 2·0$\times$10^-3^ |  |  |  |
|  | UK | 2·60 [0·95-7·09] | 0·06 |  |  |  |
|  | Czech Republic | 3·50 [0·84-14·48] | 0·08 |  |  |  |
|  | Meta-analysis | 3·63 [1·91-6·88] | 1·0$\times$10^-4^ | 4·0$\times$10^-4^ | 0·63 | 0 |
| *HLA-DQA1*05* | Scandinavia | 0·34 [0·15-0·82] | 0·02 |  |  |  |
|  | UK | 0·39 [0·15-1·04] | 0·06 |  |  |  |
|  | Czech Republic | 0·67 [0·19-2·34] | 0·53 |  |  |  |
|  | Meta-analysis | 0·41 [0·23-0·74] | 3·0$\times$10^-3^ | 7·0$\times$10^-3^ | 0·68 | 0 |
| *HLA-DQB1*05* | Scandinavia | 2·46 [1·16-5·19] | 0·02 |  |  |  |
|  | UK | 4·08 [1·79-1·29] | 8·0$\times$10^-4^ |  |  |  |
|  | Czech Republic | *N/A* | *N/A* |  |  |  |
|  | Meta-analysis | 3·09 [1·78-5·38] | 1·0$\times$10^-4^ | 4·0$\times$10^-4^ | 0·37 | 0 |
| *HLA-DQB1*06* | Scandinavia | 0·21 [0·05-0·88] | 0·03 |  |  |  |
|  | UK | 0,09 [0·01-0·69] | 0·02 |  |  |  |
|  | Czech Republic | 0·20 [0·02-1·71] | 0·14 |  |  |  |
|  | Meta-analysis | 0·16 [0·06-0·46] | 6·0$\times$10^-4^ | 2·0$\times$10^-3^ | 0·79 | 0 |
| *HLA-DRB1*01* | Scandinavia | 2·63 [1·31-5·27] | 6·0$\times$10^-3^ |  |  |  |
|  | UK | 2·42 [1·26-4·63] | 8·0$\times$10^-3^ |  |  |  |
|  | Czech Republic | 2·32 [0·82-6·53] | 0·11 |  |  |  |
|  | Meta-analysis | 2·48 [1·61-3·82] | 3·7$\times$10^-5^ | 2·2$\times$10^-4^ | 0·98 | 0 |
| *HLA-DRB1*03* | Scandinavia | 0·38 [0·16-0·90] | 0·03 |  |  |  |
|  | UK | 0·41 [0·18-0·90] | 0·03 |  |  |  |
|  | Czech Republic | 0·24 [0·07-0·88] | 0·03 |  |  |  |
|  | Meta-analysis | 0·36 [0·21-0·62] | 2·0$\times$10^-4^ | 7·5$\times$10^-4^ | 0·79 | 0 |
| *HLA-DRB1*07* | Scandinavia | 5·33 [2·09-13·62] | 5·0$\times$10^-4^ |  |  |  |
|  | UK | 2·64 [1·23-5·63] | 0·01 |  |  |  |
|  | Czech Republic | 3·05 [1·06-8·75] | 0·04 |  |  |  |
|  | Meta-analysis | 3·38 [2·02-5·65] | 4·0$\times$10^-6^ | 4·4$\times$10^-5^ | 0·51 | 0 |
| **Subgroup 8 (none)** | | | | | | |
| *HLA-DQA1*01* | Scandinavia | 1·57 [1·06-2·33] | 0·03 |  |  |  |
|  | UK | 1·53 [0·89-2·64] | 0·12 |  |  |  |
|  | Czech Republic | 1·92 [0·95-3·88] | 0·07 |  |  |  |
|  | Meta-analysis | 1·61 [1·21-2·16] | 1·0$\times$10^-3^ | 6·0$\times$10^-3^ | 0·86 | 0 |
| *HLA-DQB1*02* | Scandinavia | 0·58 [0·37-0·91] | 0·02 |  |  |  |
|  | UK | 0·45 [0·24-0·83] | 0·01 |  |  |  |
|  | Czech Republic | 0·47 [0·21-1·07] | 0·07 |  |  |  |
|  | Meta-analysis | 0·52 [0·38-0·73] | 1·0$\times$10^-4^ | 1·0$\times$10^-3^ | 0·77 | 0 |
| *HLA-DQB1*06* | Scandinavia | 1·26 [0·82-1·92] | 0·30 |  |  |  |
|  | UK | 1·97 [1·09-3·57] | 0·03 |  |  |  |
|  | Czech Republic | 2·05 [0·88-4·78] | 0·10 |  |  |  |
|  | Meta-analysis | 1·54 [1·11-2·12] | 9·0$\times$10^-3^ | 0·03 | 0·37 | 0 |
| *HLA-DRB1*13* | Scandinavia | 1·64 [1·05-2·56] | 0·03 |  |  |  |
|  | UK | 1·83 [1·14-2·93] | 0·01 |  |  |  |
|  | Czech Republic | 1·36 [0·69-2·69] | 0·37 |  |  |  |
|  | Meta-analysis | 1·65 [1·23-2·21] | 8·0$\times$10^-4^ | 0·01 | 0·79 | 0 |
| **Legend**: FDR, false discovery rate; Q, Cochran's Q test (heterogeneity across cohorts); I2, Higgins' test (degree of inconsistency and variation across the data). Pooled ORs were estimated using fixed-effect models. | | | | | | |

| **Supplementary Table 5.** Significant results for the meta-analyses for amino acids clumped by linkage disequilibrium. | | | | | | | | | | | | |
| --- | --- | --- | --- | --- | --- | --- | --- | --- | --- | --- | --- | --- |
| **Sub-groups** | **Protein:**  **position** | **AA** | **OR [95% CI]** | **P-value*** | **FDR** | **Number of variants**  **Total and grouped by p-value range** | | | | | | **Variants in LD with p<0.05**** |
|  |  |  |  |  |  | Total | >0·05 | <0·05 to 0·01 | <0·01 to 0·001 | <0·001 to 0·000 | <0·0001 |  |
| 4 | HLA-A :pos74 | H | 2·98 [1·65-5·39] | 2·94$\times$10^-4^ | 2·54$\times$10^-3^ | 22 | 0 | 3 | 8 | 11 | 0 | HLA-A_62_G; HLA-A_62_GE; HLA-A_62_GL; HLA-A_62_GR; HLA-A_62_QR; HLA-A_66; HLA-A_74_HLHLA-A-D; HLA-A_95_I; HLA-A_95_V; HLA-A_97_IM; HLA-A_97_R; HLA-A_107; HLA-A_114_H; HLA-A_114_RQ; HLA-A_116_HLHLA-A-D; HLA-A_116_Y; HLA-A_127; HLA-A_142; HLA-A_145 |
| 7 | HLA-A:pos70 |  | 2·44 [1·45-4·11] | 8·03$\times$10^-4^ | 7·51$\times$10^-3^ | 0 | 0 | 0 | 0 | 0 | 0 | NONE |
| 7 | HLA-A :pos9 | F | 2·36 [1·42-3·92] | 9·38$\times$10^-4^ | 8·41$\times$10^-3^ | 5 | 1 | 2 | 2 | 0 | 0 | HLA-A_9_FS; HLA-A_156_RL |
| 5 | HLA-B :pos9 | D | 2·23 [1·39-3·59] | 8·80$\times$10^-4^ | 1·26$\times$-01 | 10 | 0 | 6 | 4 | 0 | 0 | HLA-B_163_EL; HLA-B_163_T; HLA-B_67_F; HLA-B_67_FM |
| 6 | HLA-B :pos-8 | L | 0·43 [0·27-0·69] | 5·37$\times$10^-4^ | 4·92$\times$10^-3^ | 11 | 0 | 0 | 8 | 3 | 0 | HLA-C_156_L; HLA-C_156_LHLHLA-A-D; HLA-C_156_LQ; HLA-B_83; HLA-B_82; HLA-B_80_N; HLA-B_77_S; HLA-B_77_SG; HLA-B_-8_V; HLA-B_-11_S; HLA-B_-11_W |
| 6 | HLA-B :pos156 | Dx | 2·24 [1·43-3·5] | 4·31$\times$10^-4^ | 4·49$\times$10^-3^ | 3 | 0 | 0 | 1 | 2 | 0 | HLA-B_156_HLHLA-A-D; HLA-B_156_HLHLA-A-DW; HLA-B_156_RL |
| 6 | HLA-B :pos67 | YF | 0·46 [0·30-0·71] | 4·57$\times$10^-4^ | 4·50$\times$10^-3^ | 4 | 0 | 1 | 3 | 0 | 0 | HLA-B_67_SHLA-C; HLA-B_67_SM; HLA-B_63 |
| 6 | HLA-B :pos97 | SW | 2·49 [1·61-3·83] | 3·54$\times$10^-5^ | 2·32$\times$10^-3^ | 71 | 0 | 1 | 13 | 54 | 3 | HLA-C_339_HLA-A; HLA-C_339_T; HLA-C_326_HLA-C; HLA-C_326_S; HLA-C_307_M; HLA-C_307_V; HLA-C_306_HLA-A; HLA-C_306_V; HLA-C_305_HLA-A; HLA-C_305_T; HLA-C_295_HLA-A; HLA-C_295_V; HLA-C_285_M; HLA-C_285_ML; HLA-C_285_Mx; HLA-C_285_V; HLA-C_273_R; HLA-C_273_S; HLA-C_267_P; HLA-C_267_Q; HLA-C_261_M; HLA-C_261_V; HLA-C_253_E; HLA-C_253_Q; HLA-C_194_L; HLA-C_194_V; HLA-C_184_H; HLA-C_184_P; HLA-C_184_PR; HLA-C_184_Px; HLA-C_152_HLA-A; HLA-C_152_E; HLA-C_147; HLA-C_-9_HLA-A; HLA-C_-9_G; HLA-C_-15_I; HLA-C_-15_L; HLA-C_-17_HLA-A; HLA-C_-17_T; HLA-B_180_E; HLA-B_180_Q; HLA-B_177_HLHLA-A-D; HLA-B_177_E; HLA-B_156_LW; HLA-B_156_RHLHLA-A-D; HLA-B_116_Y; HLA-B_116_YF; HLA-B_116_YFx; HLA-B_116_Yx; HLA-B_97_RN; HLA-B_97_RT; HLA-B_97_RV; HLA-B_97_S; HLA-B_97_SN; HLA-B_97_ST; HLA-B_97_STV; HLA-B_97_STW; HLA-B_97_SV; HLA-B_97_SWN; HLA-B_97_SWV; HLA-B_74; HLA-B_45_E; HLA-B_45_EG; HLA-B_24_S; HLA-B_-10_HLA-A; HLA-B_-10_G; HLA-B_-21_M; HLA-B_-21_T; HLA-B_-23_L; HLA-B_-23_R |
| 8 | HLA-B :pos114 | DK | 1·72 [1·28-2·32] | 3·76$\times$10^-4^ | 1·78$\times$10^-2^ | 7 | 0 | 0 | 4 | 3 | 0 | HLA-B_114_HLHLA-A-D; HLA-B_114_HLHLA-A-DH; HLA-B_114_HLHLA-A-Dx; HLA-B_114_N; HLA-B_114_NH; HLA-B_114_NK; HLA-B_114_Nx |
| 8 | HLA-B :pos45 | E | 1·78 [1·29-2·43] | 3·69$\times$10^-4^ | 1·78$\times$10^-2^ | 27 | 0 | 12 | 9 | 6 | 0 | HLA-B_163_ET; HLA-B_163_L; HLA-B_97_R; HLA-B_97_RT; HLA-B_97_RV; HLA-B_97_RW; HLA-B_97_S; HLA-B_97_SN; HLA-B_97_STN; HLA-B_97_STW; HLA-B_97_SW; HLA-B_97_SWN; HLA-B_74; HLA-B_67_SM; HLA-B_45_EG |
| 8 | HLA-B :pos9 | Y | 0·56 [0·41-0·76] | 2·15$\times$10^-4^ | 1·78$\times$10^-2^ | 0 | 0 | 0 | 0 | 0 | 0 | NONE |
| 8 | HLA-B :pos95 | I | 1·91 [1·35-2·70] | 2·81$\times$10^-4^ | 1·78$\times$10^-2^ | 2 | 0 | 0 | 1 | 1 | 0 | HLA-B_95_L; HLA-B_94 |
| 6 | HLA-C :pos66 |  | 3·08 [1·93-4·9] | 2·28$\times$10^-6^ | 3·64$\times$10^-4^ | 5 | 0 | 0 | 1 | 3 | 1 | HLA-B_163_EL; HLA-B_163_T; HLA-B_67_F; HLA-B_67_FM; HLA-B_9_HLHLA-A-D |
| 8 | HLA-C :pos66 |  | 0·49 [0·34-0·70] | 8·67$\times$10^-5^ | 1·78$\times$10^-2^ | 5 | 0 | 2 | 2 | 1 | 0 | HLA-B_163_EL; HLA-B_163_T; HLA-B_9_HLHLA-A-D |
| 3 | HLA-DQB1 :pos-10 | A | 4·91 [2·75-8·78] | 7·93$\times$10^-8^ | 6·96$\times$10^-5^ | 30 | 0 | 1 | 2 | 3 | 24 | HLHLA-A-DRHLA-B1_77; HLHLA-A-DRHLA-B1_74_HLA-A; HLHLA-A-DRHLA-B1_74_HLA-AE; HLHLA-A-DRHLA-B1_74_HLA-AL; HLHLA-A-DRHLA-B1_74_R; HLHLA-A-DRHLA-B1_74_RE; HLHLA-A-DRHLA-B1_74_RQ; HLHLA-A-DRHLA-B1_73; HLHLA-A-DRHLA-B1_37_N; HLHLA-A-DRHLA-B1_37_NF; HLHLA-A-DRHLA-B1_37_SY; HLHLA-A-DRHLA-B1_26_Y; HLHLA-A-DQHLA-B1_74_HLA-A; HLHLA-A-DQHLA-B1_71_K; HLHLA-A-DQHLA-B1_71_KHLHLA-A-D; HLHLA-A-DQHLA-B1_67; HLHLA-A-DQHLA-B1_66; HLHLA-A-DQHLA-B1_57_HLA-A; HLHLA-A-DQHLA-B1_57_HLA-AS; HLHLA-A-DQHLA-B1_55_L; HLHLA-A-DQHLA-B1_52; HLHLA-A-DQHLA-B1_47; HLHLA-A-DQHLA-B1_46; HLHLA-A-DQHLA-B1_37_I; HLHLA-A-DQHLA-B1_37_Y; HLHLA-A-DQHLA-B1_30_S; HLHLA-A-DQHLA-B1_28; HLHLA-A-DQHLA-B1_-10_S; HLHLA-A-DQHLA-B1_-18_V |
| 3 | HLA-DQB1 :pos57 | AV | 0·22 [0·11-0·42] | 4·88$\times$10^-6^ | 2·86$\times$10^-4^ | 1 | 0 | 0 | 0 | 0 | 1 | HLHLA-A-DQHLA-B1_57_HLHLA-A-D |
| 3 | HLA-DQB1 :pos70 | R | 0·24 [0·12-0·47] | 3·00$\times$10^-5^ | 1·15$\times$10^-3^ | 63 | 0 | 2 | 49 | 12 | 0 | HLHLA-A-DRHLA-B1_13_SHY; HLHLA-A-DRHLA-B1_-17_HLA-A; HLHLA-A-DQHLA-A1_-16_L; HLHLA-A-DQHLA-A1_-16_M; HLHLA-A-DQHLA-A1_11_HLA-C; HLHLA-A-DQHLA-A1_11_Y; HLHLA-A-DQHLA-A1_18; HLHLA-A-DQHLA-A1_45; HLHLA-A-DQHLA-A1_47_R; HLHLA-A-DQHLA-A1_48; HLHLA-A-DQHLA-A1_50_E; HLHLA-A-DQHLA-A1_52_S; HLHLA-A-DQHLA-A1_53_K; HLHLA-A-DQHLA-A1_55; HLHLA-A-DQHLA-A1_56_G; HLHLA-A-DQHLA-A1_61; HLHLA-A-DQHLA-A1_64; HLHLA-A-DQHLA-A1_66; HLHLA-A-DQHLA-A1_69_HLA-A; HLHLA-A-DQHLA-A1_69_L; HLHLA-A-DQHLA-A1_76_M; HLHLA-A-DQHLA-A1_80; HLHLA-A-DQHLA-A1_175_Q; HLHLA-A-DQHLA-A1_175_Qx; HLHLA-A-DQHLA-A1_218_Q; HLHLA-A-DQHLA-A1_218_R; HLHLA-A-DQHLA-B1_221_H; HLHLA-A-DQHLA-B1_221_Q; HLHLA-A-DQHLA-B1_220_H; HLHLA-A-DQHLA-B1_220_R; HLHLA-A-DQHLA-B1_203_I; HLHLA-A-DQHLA-B1_203_V; HLHLA-A-DQHLA-B1_125_HLA-A; HLHLA-A-DQHLA-B1_125_HLA-Ax; HLHLA-A-DQHLA-B1_90_I; HLHLA-A-DQHLA-B1_90_T; HLHLA-A-DQHLA-B1_89_G; HLHLA-A-DQHLA-B1_89_T; HLHLA-A-DQHLA-B1_87_L; HLHLA-A-DQHLA-B1_87_Lx; HLHLA-A-DQHLA-B1_86_HLA-A; HLHLA-A-DQHLA-B1_86_E; HLHLA-A-DQHLA-B1_86_EG; HLHLA-A-DQHLA-B1_86_Ex; HLHLA-A-DQHLA-B1_85_L; HLHLA-A-DQHLA-B1_85_V; HLHLA-A-DQHLA-B1_84_E; HLHLA-A-DQHLA-B1_84_Q; HLHLA-A-DQHLA-B1_70_G; HLHLA-A-DQHLA-B1_55_R; HLHLA-A-DQHLA-B1_53; HLHLA-A-DQHLA-B1_-4_L; HLHLA-A-DQHLA-B1_-4_V; HLHLA-A-DQHLA-B1_-5_P; HLHLA-A-DQHLA-B1_-5_Px; HLHLA-A-DQHLA-B1_-6_S; HLHLA-A-DQHLA-B1_-6_T; HLHLA-A-DQHLA-B1_-6_THLA-A; HLHLA-A-DQHLA-B1_-6_Tx; HLHLA-A-DQHLA-B1_-21_HLHLA-A-D; HLHLA-A-DQHLA-B1_-21_G |
| 6 | HLA-DQB1 :pos-10 | S | 2·6 [1·57-4·33] | 2·27$\times$10^-4^ | 2·71$\times$10^-3^ | 29 | 4 | 2 | 11 | 12 | 0 | HLHLA-A-DRHLA-B1_74_RQ; HLHLA-A-DRHLA-B1_73; HLHLA-A-DQHLA-A1_75; HLHLA-A-DQHLA-A1_107_I; HLHLA-A-DQHLA-A1_107_T; HLHLA-A-DQHLA-A1_156_F; HLHLA-A-DQHLA-A1_156_L; HLHLA-A-DQHLA-A1_161_HLHLA-A-D; HLHLA-A-DQHLA-A1_161_E; HLHLA-A-DQHLA-A1_163_I; HLHLA-A-DQHLA-A1_163_S; HLHLA-A-DQHLA-A1_175_K; HLHLA-A-DQHLA-A1_175_QE; HLHLA-A-DQHLA-B1_74_HLA-A; HLHLA-A-DQHLA-B1_71_K; HLHLA-A-DQHLA-B1_55_L; HLHLA-A-DQHLA-B1_52; HLHLA-A-DQHLA-B1_47; HLHLA-A-DQHLA-B1_46; HLHLA-A-DQHLA-B1_37_I; HLHLA-A-DQHLA-B1_37_Y; HLHLA-A-DQHLA-B1_30_S; HLHLA-A-DQHLA-B1_28 |
| 7 | HLA-DQB1 :pos135 | G | 4·6 [2·08-10·16] | 1·63$\times$10^-4^ | 2·65$\times$10^-3^ | 26 | 0 | 0 | 15 | 11 | 0 | HLHLA-A-DRHLA-B1_181_M; HLHLA-A-DRHLA-B1_78; HLHLA-A-DRHLA-B1_74_Q; HLHLA-A-DRHLA-B1_60_S; HLHLA-A-DRHLA-B1_57_V; HLHLA-A-DRHLA-B1_37_F; HLHLA-A-DRHLA-B1_37_FL; HLHLA-A-DRHLA-B1_30_L; HLHLA-A-DRHLA-B1_30_LG; HLHLA-A-DRHLA-B1_30_LH; HLHLA-A-DRHLA-B1_30_LR; HLHLA-A-DRHLA-B1_30_YHLA-C; HLHLA-A-DRHLA-B1_30_YHLA-CG; HLHLA-A-DRHLA-B1_30_YHLA-CH; HLHLA-A-DRHLA-B1_30_YHLA-CR; HLHLA-A-DRHLA-B1_25; HLHLA-A-DRHLA-B1_14; HLHLA-A-DRHLA-B1_13_Y; HLHLA-A-DRHLA-B1_11_G; HLHLA-A-DRHLA-B1_11_GHLHLA-A-D; HLHLA-A-DRHLA-B1_4_Q; HLHLA-A-DRHLA-B1_4_R; HLHLA-A-DQHLA-A1_47_K; HLHLA-A-DQHLA-A1_52_H; HLHLA-A-DQHLA-A1_54; HLHLA-A-DQHLA-B1_135_HLHLA-A-D |
| 7 | HLA-DQB1 :pos57 | D | 0·32 [0·17-0·62] | 6·63$\times$10^-4^ | 6·47$\times$10^-3^ | 3 | 2 | 0 | 1 | 0 | 0 | HLHLA-A-DQHLA-B1_57_HLA-AV |
| 7 | HLA-DQB1 :pos74 | S | 3·28 [1·89-5·70] | 2·59$\times$10^-5^ | 7·60$\times$10^-4^ | 21 | 0 | 0 | 1 | 6 | 14 | HLHLA-A-DRHLA-B1_13_FG; HLHLA-A-DQHLA-B1_224_Q; HLHLA-A-DQHLA-B1_224_R; HLHLA-A-DQHLA-B1_125_HLA-AG; HLHLA-A-DQHLA-B1_125_S; HLHLA-A-DQHLA-B1_116_I; HLHLA-A-DQHLA-B1_116_V; HLHLA-A-DQHLA-B1_87_LF; HLHLA-A-DQHLA-B1_87_Y; HLHLA-A-DQHLA-B1_71_HLA-A; HLHLA-A-DQHLA-B1_71_KT; HLHLA-A-DQHLA-B1_26_G; HLHLA-A-DQHLA-B1_26_L; HLHLA-A-DQHLA-B1_14_L; HLHLA-A-DQHLA-B1_14_M; HLHLA-A-DQHLA-B1_-5_PL; HLHLA-A-DQHLA-B1_-5_S; HLHLA-A-DQHLA-B1_-9_I; HLHLA-A-DQHLA-B1_-9_M; HLHLA-A-DQHLA-B1_-27_HLA-A; HLHLA-A-DQHLA-B1_-27_S |
| 8 | HLA-DQB1 :pos57 | AS | 0·57 [0·41-0·79] | 7·78$\times$10^-4^ | 1·92$\times$10^-2^ | 7 | 2 | 4 | 1 | 0 | 0 | HLHLA-A-DQHLA-B1_57_HLA-A |
| 2 | HLA-DRB1 :pos13 | SYF | 1·95 [1·34-2·86] | 5·29$\times$10^-4^ | 1·24$\times$10^-1^ | 11 | 0 | 4 | 6 | 1 | 0 | HLHLA-A-DRHLA-B1_96_HE; HLHLA-A-DRHLA-B1_96_QY; HLHLA-A-DRHLA-B1_13_RH; HLHLA-A-DRHLA-B1_13_SF; HLHLA-A-DRHLA-B1_11_PV; HLHLA-A-DRHLA-B1_11_SGL; HLHLA-A-DQHLA-B1_30_Y |
| 3 | HLA-DRB1 :pos26 | F | 2·49 [1·48-4·16] | 5·34$\times$10^-4^ | 1·07$\times$10^-2^ | 3 | 0 | 0 | 3 | 0 | 0 | HLHLA-A-DQHLA-B1_77; HLHLA-A-DQHLA-B1_71_KHLA-A; HLHLA-A-DQHLA-B1_38 |
| 3 | HLA-DRB1 :pos37 | NL | 2·67 [1·62-4·43] | 1·31$\times$10^-4^ | 3·36$\times$10^-3^ | 2 | 0 | 0 | 1 | 1 | 0 | HLHLA-A-DRHLA-B1_71_KE; HLHLA-A-DRHLA-B1_32 |
| 3 | HLA-DRB1 :pos67 | L | 0·33 [0·19-0·59] | 1·34$\times$10^-4^ | 3·36$\times$10^-3^ | 2 | 0 | 2 | 0 | 0 | 0 | NONE |
| 3 | HLA-DRB1 :pos71 | K | 2·75 [1·62-4·67] | 1·83$\times$10^-4^ | 4·27$\times$10^-3^ | 2 | 0 | 1 | 0 | 1 | 0 | HLHLA-A-DRHLA-B1_74_RL |
| 4 | HLA-DRB1 :pos13 | HY | 3·65 [1·99-6·67] | 2·77$\times$10^-5^ | 4·70$\times$10^-4^ | 32 | 16 | 0 | 2 | 2 | 12 | HLHLA-A-DRHLA-B1_104_HLA-A; HLHLA-A-DRHLA-B1_104_S; HLHLA-A-DRHLA-B1_98_E; HLHLA-A-DRHLA-B1_98_K; HLHLA-A-DRHLA-B1_37_NS; HLHLA-A-DRHLA-B1_37_YF; HLHLA-A-DRHLA-B1_13_SFG; HLHLA-A-DRHLA-B1_13_SRF; HLHLA-A-DRHLA-B1_11_SLHLHLA-A-D; HLHLA-A-DRHLA-B1_11_SPL; HLHLA-A-DRHLA-B1_11_VG; HLHLA-A-DQHLA-A1_47_RHLA-C; HLHLA-A-DQHLA-A1_50_L; HLHLA-A-DQHLA-A1_53_R; HLHLA-A-DQHLA-A1_215_F; HLHLA-A-DQHLA-A1_215_L |
| 4 | HLA-DRB1 :pos14 |  | 6·37 [2·95-13·74] | 2·41$\times$10^-6^ | 2·29$\times$10^-4^ | 33 | 0 | 0 | 0 | 2 | 31 | HLHLA-A-DRHLA-B1_181_M; HLHLA-A-DRHLA-B1_181_T; HLHLA-A-DRHLA-B1_78; HLHLA-A-DRHLA-B1_74_Q; HLHLA-A-DRHLA-B1_74_QE; HLHLA-A-DRHLA-B1_74_QL; HLHLA-A-DRHLA-B1_60_S; HLHLA-A-DRHLA-B1_60_Y; HLHLA-A-DRHLA-B1_57_HLHLA-A-DS; HLHLA-A-DRHLA-B1_57_V; HLHLA-A-DRHLA-B1_37_F; HLHLA-A-DRHLA-B1_37_FL; HLHLA-A-DRHLA-B1_30_L; HLHLA-A-DRHLA-B1_30_LG; HLHLA-A-DRHLA-B1_30_LH; HLHLA-A-DRHLA-B1_30_LR; HLHLA-A-DRHLA-B1_30_YHLA-C; HLHLA-A-DRHLA-B1_30_YHLA-CG; HLHLA-A-DRHLA-B1_30_YHLA-CH; HLHLA-A-DRHLA-B1_30_YHLA-CR; HLHLA-A-DRHLA-B1_25; HLHLA-A-DRHLA-B1_13_Y; HLHLA-A-DRHLA-B1_13_YG; HLHLA-A-DRHLA-B1_11_G; HLHLA-A-DRHLA-B1_11_GHLHLA-A-D; HLHLA-A-DRHLA-B1_4_Q; HLHLA-A-DRHLA-B1_4_R; HLHLA-A-DQHLA-A1_25; HLHLA-A-DQHLA-A1_47_K; HLHLA-A-DQHLA-A1_52_H; HLHLA-A-DQHLA-A1_54; HLHLA-A-DQHLA-B1_135_HLHLA-A-D; HLHLA-A-DQHLA-B1_135_G |
| 4 | HLA-DRB1 :pos30 | YHG | 3·97 [2·19-7·18] | 5·30$\times$10^-6^ | 2·29$\times$10^-4^ | 18 | 5 | 0 | 0 | 0 | 13 | HLHLA-A-DRHLA-B1_30_LHLA-C; HLHLA-A-DRHLA-B1_30_Y; HLHLA-A-DRHLA-B1_30_YG; HLHLA-A-DRHLA-B1_30_YGR; HLHLA-A-DRHLA-B1_30_YH; HLHLA-A-DRHLA-B1_30_YHR; HLHLA-A-DRHLA-B1_30_YR; HLHLA-A-DRHLA-B1_28_HLHLA-A-D; HLHLA-A-DRHLA-B1_28_E; HLHLA-A-DRHLA-B1_13_SRH; HLHLA-A-DRHLA-B1_13_YF; HLHLA-A-DRHLA-B1_11_GL; HLHLA-A-DRHLA-B1_11_SPV |
| 4 | HLA-DRB1 :pos37 | NL | 0·18 [0·08-0·43] | 8·80$\times$10^-5^ | 1·13$\times$10^-3^ | 9 | 3 | 0 | 0 | 6 | 0 | HLHLA-A-DRHLA-B1_77; HLHLA-A-DRHLA-B1_74_R; HLHLA-A-DRHLA-B1_74_RE; HLHLA-A-DRHLA-B1_37_N; HLHLA-A-DRHLA-B1_32; HLHLA-A-DRHLA-B1_26_Y |
| 4 | HLA-DRB1 :pos37 | SF | 3·05 [1·66-5·62] | 3·46$\times$10^-4^ | 2·87$\times$10^-3^ | 21 | 8 | 0 | 7 | 6 | 0 | HLHLA-A-DRHLA-B1_140_HLA-A; HLHLA-A-DRHLA-B1_140_T; HLHLA-A-DRHLA-B1_37_NY; HLHLA-A-DRHLA-B1_13_RY; HLHLA-A-DRHLA-B1_13_SH; HLHLA-A-DRHLA-B1_13_SHG; HLHLA-A-DRHLA-B1_11_PG; HLHLA-A-DRHLA-B1_11_SV; HLHLA-A-DRHLA-B1_11_SVHLHLA-A-D; HLHLA-A-DRHLA-B1_9_E; HLHLA-A-DRHLA-B1_9_W; HLHLA-A-DQHLA-A1_47_RK; HLHLA-A-DQHLA-A1_52_R |
| 4 | HLA-DRB1 :pos47 |  | 5·48 [2·71-11·1] | 2·10$\times$10^-6^ | 2·29$\times$10^-4^ | 9 | 1 | 1 | 0 | 0 | 7 | HLHLA-A-DRHLA-B1_13_SR; HLHLA-A-DRHLA-B1_13_SRG; HLHLA-A-DRHLA-B1_11_SP; HLHLA-A-DRHLA-B1_11_SPHLHLA-A-D; HLHLA-A-DQHLA-A1_34; HLHLA-A-DQHLA-A1_175_E; HLHLA-A-DQHLA-A1_175_QK |
| 4 | HLA-DRB1 :pos57 | DA | 3·67 [1·88-7·15] | 1·35$\times$10^-4^ | 1·55$\times$10^-3^ | 2 | 0 | 0 | 0 | 2 | 0 | HLHLA-A-DRHLA-B1_74_RHLA-A; HLHLA-A-DRHLA-B1_57_HLHLA-A-D |
| 4 | HLA-DRB1 :pos74 | RL | 0·15 [0·06-0·40] | 1·41$\times$10^-4^ | 1·59$\times$10^-3^ | 36 | 18 | 1 | 0 | 17 | 0 | HLHLA-A-DRHLA-B1_74_HLA-AQ; HLHLA-A-DQHLA-A1_40; HLHLA-A-DQHLA-A1_47_HLA-C; HLHLA-A-DQHLA-A1_50_V; HLHLA-A-DQHLA-A1_51; HLHLA-A-DQHLA-A1_53_Q; HLHLA-A-DQHLA-A1_75; HLHLA-A-DQHLA-A1_107_I; HLHLA-A-DQHLA-A1_107_T; HLHLA-A-DQHLA-A1_156_F; HLHLA-A-DQHLA-A1_156_L; HLHLA-A-DQHLA-A1_161_HLHLA-A-D; HLHLA-A-DQHLA-A1_161_E; HLHLA-A-DQHLA-A1_163_I; HLHLA-A-DQHLA-A1_163_S; HLHLA-A-DQHLA-A1_175_K; HLHLA-A-DQHLA-A1_175_QE |
| 4 | HLA-DRB1 :pos86 |  | 5·97 [2·86-12·5] | 2·02$\times$10^-6^ | 2·29$\times$10^-4^ | 0 | 0 | 0 | 0 | 0 | 0 | NONE |
| 5 | HLA-DRB1 :pos37 | N | 1·98 [1·32-2·97] | 9·21$\times$10^-4^ | 1·26$\times$10^-1^ | 11 | 2 | 2 | 7 | 0 | 0 | HLHLA-A-DRHLA-B1_77; HLHLA-A-DRHLA-B1_74_R; HLHLA-A-DRHLA-B1_74_RE; HLHLA-A-DRHLA-B1_71_KE; HLHLA-A-DRHLA-B1_37_NL; HLHLA-A-DRHLA-B1_32; HLHLA-A-DRHLA-B1_26_Y |
| 6 | HLA-DRB1 :pos11 | SPD | 0·31 [0·18-0·53] | 2·09$\times$10^-5^ | 1·60$\times$10^-3^ | 22 | 0 | 10 | 5 | 5 | 2 | HLHLA-A-DRHLA-B1_96_HQ; HLHLA-A-DRHLA-B1_96_YE; HLHLA-A-DRHLA-B1_47; HLHLA-A-DRHLA-B1_13_HF; HLHLA-A-DRHLA-B1_13_HY; HLHLA-A-DRHLA-B1_13_SR; HLHLA-A-DRHLA-B1_13_SRG; HLHLA-A-DRHLA-B1_11_SP; HLHLA-A-DRHLA-B1_11_SPG; HLHLA-A-DRHLA-B1_11_VG; HLHLA-A-DRHLA-B1_11_VL; HLHLA-A-DQHLA-A1_34 |
| 6 | HLA-DRB1 :pos30 | YG | 0·25 [0·11-0·54] | 4·38$\times$10^-4^ | 4·49$\times$10^-3^ | 18 | 0 | 4 | 11 | 3 | 0 | HLHLA-A-DRHLA-B1_30_LHLA-C; HLHLA-A-DRHLA-B1_30_Y; HLHLA-A-DRHLA-B1_30_YGR; HLHLA-A-DRHLA-B1_30_YH; HLHLA-A-DRHLA-B1_30_YHG; HLHLA-A-DRHLA-B1_30_YHR; HLHLA-A-DRHLA-B1_30_YLG; HLHLA-A-DRHLA-B1_30_YR; HLHLA-A-DRHLA-B1_28_HLHLA-A-D; HLHLA-A-DRHLA-B1_28_E; HLHLA-A-DRHLA-B1_13_SRH; HLHLA-A-DRHLA-B1_13_YF; HLHLA-A-DRHLA-B1_11_GL; HLHLA-A-DRHLA-B1_11_SPV |
| 6 | HLA-DRB1 :pos37 | N | 2·62 [1·69-4·06] | 1·74$\times$10^-5^ | 1·45$\times$10^-3^ | 7 | 0 | 1 | 2 | 2 | 2 | HLHLA-A-DRHLA-B1_71_KE; HLHLA-A-DRHLA-B1_37_NF; HLHLA-A-DRHLA-B1_37_NL; HLHLA-A-DRHLA-B1_37_SY; HLHLA-A-DRHLA-B1_32; HLHLA-A-DQHLA-B1_-10_HLA-A |
| 6 | HLA-DRB1 :pos71 | KA | 0·35 [0·22-0·55] | 3·76$\times$10^-6^ | 4·05$\times$10^-4^ | 2 | 0 | 0 | 0 | 1 | 1 | HLHLA-A-DRHLA-B1_71_K; HLHLA-A-DRHLA-B1_71_R |
| 6 | HLA-DRB1 :pos74 | AQ | 3·16 [1·99-5·02] | 1·02$\times$10^-6^ | 3·55$\times$10^-4^ | 16 | 0 | 0 | 0 | 11 | 5 | HLHLA-A-DRHLA-B1_77; HLHLA-A-DRHLA-B1_74_HLA-A; HLHLA-A-DRHLA-B1_74_HLA-AE; HLHLA-A-DRHLA-B1_74_HLA-AL; HLHLA-A-DRHLA-B1_74_R; HLHLA-A-DRHLA-B1_74_RE; HLHLA-A-DRHLA-B1_74_RL; HLHLA-A-DRHLA-B1_26_Y; HLHLA-A-DQHLA-A1_40; HLHLA-A-DQHLA-A1_47_HLA-C; HLHLA-A-DQHLA-A1_50_V; HLHLA-A-DQHLA-A1_51; HLHLA-A-DQHLA-A1_53_Q; HLHLA-A-DQHLA-B1_71_KHLHLA-A-D; HLHLA-A-DQHLA-B1_67; HLHLA-A-DQHLA-B1_66 |
| 6 | HLA-DRB1 :pos86 |  | 0·42 [0·27-0·66] | 1·79$\times$10^-4^ | 2·52$\times$10^-3^ | 0 | 0 | 0 | 0 | 0 | 0 | NONE |
| 7 | HLA-DRB1 :pos11 | GL | 4·45 [2·62-7·56] | 3·57$\times$10^-8^ | 1·14$\times$10^-5^ | 18 | 0 | 0 | 0 | 0 | 18 | HLHLA-A-DRHLA-B1_96_E; HLHLA-A-DRHLA-B1_96_Ex; HLHLA-A-DRHLA-B1_30_HLA-C; HLHLA-A-DRHLA-B1_30_HLA-CR; HLHLA-A-DRHLA-B1_30_LHLA-C; HLHLA-A-DRHLA-B1_30_Y; HLHLA-A-DRHLA-B1_30_YG; HLHLA-A-DRHLA-B1_30_YGR; HLHLA-A-DRHLA-B1_30_YH; HLHLA-A-DRHLA-B1_30_YHG; HLHLA-A-DRHLA-B1_30_YHR; HLHLA-A-DRHLA-B1_30_YR; HLHLA-A-DRHLA-B1_28_HLHLA-A-D; HLHLA-A-DRHLA-B1_28_E; HLHLA-A-DRHLA-B1_13_SRH; HLHLA-A-DRHLA-B1_13_YF; HLHLA-A-DRHLA-B1_11_L; HLHLA-A-DRHLA-B1_11_SPV |
| 7 | HLA-DRB1 :pos11 | SVD | 2·66 [1·59-4·45] | 2·00$\times$10^-4^ | 2·92$\times$10^-3^ | 22 | 11 | 2 | 0 | 9 | 0 | HLA-DRB1_140_A; HLA-DRB1_140_T; HLA-DRB1_37_NY; HLA-DRB1_37_SF; HLA-DRB1_13_SH; HLA-DRB1_13_SHG; HLA-DRB1_11_SV; HLA-DRB1_9_E; HLA-DRB1_9_W |
| 7 | HLA-DRB1 :pos30 | CH | 2·49 [1·48-4·16] | 6·53$\times$10^-5^ | 1·30$\times$10^-3^ | 12 | 0 | 0 | 0 | 4 | 8 | HLA-DRB1_31_F; HLA-DRB1_31_I; HLA-DRB1_30_CG; HLA-DRB1_30_YL; HLA-DRB1_30_YLG; HLA-DRB1_30_YLH; HLA-DRB1_30_YLR; HLA-DRB1_26_L; HLA-DRB1_13_F; HLA-DRB1_11_LHLA-D; HLA-DQB1_57_AHLA-D; HLA-DQB1_57_V |
| 7 | HLA-DRB1 :pos37 | NL | 0·32 [0·17-0·60] | 3·88$\times$10^-4^ | 4·61$\times$10^-3^ | 8 | 2 | 1 | 3 | 2 | 0 | HLA-DRB1_77; HLA-DRB1_74_R; HLA-DRB1_37_N; HLA-DRB1_32; HLA-DRB1_26_Y |
| 7 | HLA-DRB1 :pos47 |  | 5·19 [2·81-9·58] | 1·42$\times$10^-7^ | 1·24$\times$10^-5^ | 9 | 0 | 0 | 3 | 1 | 5 | HLA-DRB1_13_SR; HLA-DRB1_13_SRG; HLA-DRB1_13_SRY; HLA-DRB1_11_SP; HLA-DRB1_11_SPHLA-D; HLA-DRB1_11_SPG; HLA-DQA1_34; HLA-DQA1_175_E; HLA-DQA1_175_QK |
| 7 | HLA-DRB1 :pos71 | K | 0·31 [0·16-0·59] | 4·32$\times$10^-4^ | 5·00$\times$10^-3^ | 1 | 1 | 0 | 0 | 0 | 0 | NONE |
| 7 | HLA-DRB1 :pos71 | R | 5·58 [3·07-10·1] | 1·60$\times$10^-8^ | 1·14$\times$10^-5^ | 2 | 0 | 0 | 0 | 0 | 2 | HLA-DRB1_71_KA; HLA-DRB1_71_KE |
| 7 | HLA-DRB1 :pos74 | QL | 3·43 [1·78-6·62] | 2·42$\times$10^-4^ | 3·32$\times$10^-3^ | 5 | 0 | 2 | 2 | 1 | 0 | HLA-DRB1_181_T; HLA-DRB1_74_RA; HLA-DRB1_13_YG |
| 7 | HLA-DRB1 :pos86 |  | 2·49 [1·45-4·28] | 9·25$\times$10^-4^ | 8·38$\times$10^-3^ | 0 | 0 | 0 | 0 | 0 | 0 | NONE |
| 7 | HLA-DRB1 :pos96 | HQ | 2·56 [1·50-4·38] | 5·82$\times$10^-4^ | 5·88$\times$10^-3^ | 14 | 11 | 0 | 1 | 2 | 0 | HLA-DRB1_96_YE; HLA-DRB1_13_HF; HLA-DRB1_11_VL |
| 8 | HLA-DRB1 :pos71 | K | 0·53 [0·38-0·75] | 2·82$\times$10^-4^ | 1·78$\times$10^-2^ | 2 | 1 | 0 | 1 | 0 | 0 | HLA-DRB1_71_KA |
| 8 | HLA-DRB1 :pos74 | A | 0·52 [0·37-0·72] | 1·07$\times$10^-4^ | 1·78$\times$10^-2^ | 25 | 0 | 0 | 4 | 21 | 0 | HLA-DRB1_77; HLA-DRB1_74_AE; HLA-DRB1_74_AL; HLA-DRB1_74_AQ; HLA-DRB1_74_R; HLA-DRB1_74_RE; HLA-DRB1_74_RL; HLA-DRB1_74_RQ; HLA-DRB1_73; HLA-DRB1_26_Y; HLA-DQB1_74_A; HLA-DQB1_71_K; HLA-DQB1_71_KHLA-D; HLA-DQB1_67; HLA-DQB1_66; HLA-DQB1_55_L; HLA-DQB1_52; HLA-DQB1_47; HLA-DQB1_46; HLA-DQB1_37_I; HLA-DQB1_37_Y; HLA-DQB1_30_S; HLA-DQB1_28; HLA-DQB1_-10_A; HLA-DQB1_-10_S |
| **Legend:**  *Significance threshold for index variants: p < 0.001, linkage disequilibrium of r2>0.5.  **The first term is the protein, the second is the position and the third is the amino acid.  ^†^Autoantibodies dominating each subgroup: 1: Anti-Ro52, 2: Anti-U1RNP, 3: Anti-PM/Scl, 4: Anti-Mi2, 5: Anti-Jo1, 6: Anti-Jo1/Ro52, 7: Anti-TIF1$\gamma$, 8: negative for autoantibodies tested | | | | | | | | | | | | |

| **Supplementary Table 6.** Conditional analyses for class II alleles, including UK, Scandinavia, and Czech Republic populations. | | | | | |
| --- | --- | --- | --- | --- | --- |
| **Subgroups** | **Conditional on** | **Independent Signals** | | | |
|  |  | **Alleles** | **OR [95% CI]** | **P-value** | **FDR** |
| 1 | HLA-DRB1*03 | HLA-DRB1*15 | 1·87 [1·05-3·34] | 0·034 | 0·45 |
| 2 | HLA-DRB1*15 | HLA-DRB1*03 | 0·38 [0·24-0·63] | 1·59$\times$10^-4^ | 2·80$\times$10^-3^ |
|  |  | HLA-DQB1*03 | 2·78 [1·61-4·82] | 2·67$\times$10^-4^ | 2·80$\times$10^-3^ |
|  |  | HLA-DRB1*11 | 2·60 [1·48-4·60] | 9·72$\times$10^-4^ | 5·22$\times$10^-3^ |
|  |  | HLA-DRB1*04 | 2·10 [1·34-3·15] | 9·94$\times$10^-4^ | 5·22$\times$10^-3^ |
|  |  | HLA-DQA1*03 | 2·54 [1·39-4·65] | 2·38$\times$10^-3^ | 8·92$\times$10^-3^ |
|  |  | HLA-DQB1*02 | 0·39 [0·22-0·72] | 2·55$\times$10^-3^ | 8·92$\times$10^-3^ |
|  | HLA-DRB1*15, HLA-DRB1*03 | HLA-DQB1*03 | 2·26 [1·26-4·00] | 6·08$\times$10^-3^ | 0·12 |
| 3 | HLA-DQB1*02 | HLA-DRB1*04 | 3·84 [1·65-8·95] | 1·82$\times$10^-3^ | 0·03 |
|  | HLA-DRB1*03 | HLA-DRB1*04 | 2·50 [1·37-4·56] | 2·88$\times$10^-3^ | 0·05 |
|  |  | HLA-DQB1*02 | 4.00 [1·39-11·3] | 9·81$\times$10^-3^ | 0·08 |
| 4 | HLA-DRB1*07 | HLA-DRB1*03 | 0·22 [0·08-0·58] | 2·58$\times$10^-3^ | 0·05 |
|  |  | HLA-DQB1*02 | 0·34 [0·26-0·72] | 5·14$\times$10^-3^ | 0·05 |
|  |  | HLA-DQB1*05 | 2·52 [1·29-4·92] | 6·92$\times$10^-3^ | 0·05 |
|  |  | HLA-DRB1*01 | 2·39 [1·27-4·62] | 9·53$\times$10^-3^ | 0·05 |
|  |  | HLA-DQA1*01 | 2·49 [1·22-5·10] | 0·01 | 0·05 |
|  |  | HLA-DRB1*16 | 4·35 [1·33-14·2] | 0·02 | 0·05 |
| 5 | HLA-DRB1*03 | HLA-DQB1*02 | 0·19 [0·05-0·79] | 0·02 | 0·45 |
| 6 | HLA-DRB1*03 | HLA-DQB1*02 | 0·25 [0·07-0·89] | 0·03 | 0·48 |
| 7 | HLA-DRB1*07 | HLA-DRB1*01 | 3.00 [1·68-5·36] | 2·11$\times$10^-4^ | 4·65$\times$10^-3^ |
|  |  | HLA-DQB1*05 | 2·70 [1·37-5·27] | 3·94$\times$10^-3^ | 0·04 |
|  | HLA-DQA1*02 | HLA-DRB1*01 | 3·79 [1·79-8·02] | 4·95$\times$10^-4^ | 0·01 |
|  |  | HLA-DQB1*05 | 2·74 [1·39-5·32] | 3·60$\times$10^-3^ | 0·04 |
|  |  | HLA-DQB1*06 | 0·15 [0·04-0·67] | 0·01 | 0·09 |
|  | HLA-DRB1*01 | HLA-DRB1*07 | 3·60 [1·90-6·91] | 9·24$\times$10^-5^ | 1·94$\times$10^-3^ |
|  |  | HLA-DQA1*02 | 3·60 [1·50-8·56] | 4·13$\times$10^-3^ | 0·03 |
|  |  | HLA-DRB1*03 | 0·40 [0·19-0·73] | 4·45$\times$10^-3^ | 0·03 |
|  | HLA-DRB1*07, HLA-DRB1*01 | HLA-DRB1*04 | 2·60 [1·28-5·36] | 8·54$\times$10^-3^ | 0·17 |
| 8 | HLA-DQA1*01 | HLA-DRB1*11 | 3·40 [1·56-7·92] | 2·07$\times$10^-3^ | 0·04 |
|  |  | HLA-DQB1*03 | 1·59 [0·85-2·97] | 0·02 | 0·13 |
|  | HLA-DQB1*06 | HLA-DQB1*02 | 0·78 [0·40-1·51] | 8·39$\times$10^-3^ | 0·11 |
|  |  | HLA-DRB1*03 | 0·51 [0·30-0·86] | 0·01 | 0·11 |
|  | HLA-DRB1*13 | HLA-DQB1*02 | 0·65 [0·33-0·34] | 2·16$\times$10^-3^ | 0·05 |
|  |  | HLA-DRB1*14 | 2·93 [1·32-6·53] | 8·46$\times$10^-3^ | 0·06 |
|  |  | HLA-DQB1*06 | 2·10 [1·20-3·60] | 8·86$\times$10^-3^ | 0·06 |
|  | HLADQA1*01, HLA-DRB1*11 | HLA-DRB1*04 | 2·50 [1·26-4·79] | 8·42$\times$10^-3^ | 0·16 |
| **Legend**: Autoantibodies dominating each subgroup: 1: Anti-Ro52, 2: Anti-U1RNP, 3: Anti-PM/Scl, 4: Anti-Mi2, 5: Anti-Jo1, 6: Anti-Jo1/Ro52, 7: Anti-TIF1$\gamma$, 8: negative for autoantibodies tested. | | | | | |

**References**

1. Eriksson D, Bianchi M, Landegren N, et al. Extended exome sequencing identifies BACH2 as a novel major risk locus for Addison's disease. *J Intern Med* 2016; **280**(6): 595-608.

2. Sandling JK, Pucholt P, Hultin Rosenberg L, et al. Molecular pathways in patients with systemic lupus erythematosus revealed by gene-centred DNA sequencing. *Ann Rheum Dis* 2021; **80**(1): 109-17.

3. Bianchi M, Kozyrev SV, Notarnicola A, et al. Contribution of Rare Genetic Variation to Disease Susceptibility in a Large Scandinavian Myositis Cohort. *Arthritis Rheumatol* 2022; **74**(2): 342-52.

4. Eriksson D, Bianchi M, Landegren N, et al. Extended exome sequencing identifies BACH2 as a novel major risk locus for Addison's disease. *J Intern Med* 2016; **280**(6): 595-608.

5. Li H, Durbin R. Fast and accurate long-read alignment with Burrows-Wheeler transform. *Bioinformatics* 2010; **26**(5): 589-95.

6. McKenna A, Hanna M, Banks E, et al. The Genome Analysis Toolkit: a MapReduce framework for analyzing next-generation DNA sequencing data. *Genome Res* 2010; **20**(9): 1297-303.

7. Van der Auwera GA, Carneiro MO, Hartl C, et al. From FastQ data to high confidence variant calls: the Genome Analysis Toolkit best practices pipeline. *Curr Protoc Bioinformatics* 2013; **43**: 11 0 1- 0 33.

8. DePristo MA, Banks E, Poplin R, et al. A framework for variation discovery and genotyping using next-generation DNA sequencing data. *Nat Genet* 2011; **43**(5): 491-8.

9. Jia X, Han B, Onengut-Gumuscu S, et al. Imputing amino acid polymorphisms in human leukocyte antigens. *PLoS One* 2013; **8**(6): e64683.

10. Purcell SN, B. Todd-Brown, K. Thomas, L. Ferreira, MAR. Bender, D. Maller, J. Sklar, P. de Bakker, PIW. Daly, MJ. Sham, PC. PLINK: a toolset for whole-genome association and population-based linkage analysis  *American Journal of Human Genetics* 2007; **81**.

11. R Core Team. R: A language and environment for statistical computing. R Foundation for Statistical Computing; 2019.

12. Maechler M, Rousseeuw P, Struyf A, Hubert M, Hornik K. cluster: Cluster Analysis Basics and Extensions2021. (accessed.

13. Venables WN, Ripley BD. Modern Applied Statistics with S. Fourth ed: Springer; 2002.

14. Balduzzi S, Rucker G, Schwarzer G. How to perform a meta-analysis with R: a practical tutorial. *Evid Based Ment Health* 2019; **22**(4): 153-60.

15. Lilleker JB, Vencovsky J, Wang G, et al. The EuroMyositis registry: an international collaborative tool to facilitate myositis research. *Ann Rheum Dis* 2018; **77**(1): 30-9.

16. Rothwell S, Cooper RG, Lundberg IE, et al. Dense genotyping of immune-related loci in idiopathic inflammatory myopathies confirms HLA alleles as the strongest genetic risk factor and suggests different genetic background for major clinical subgroups. *Ann Rheum Dis* 2016; **75**(8): 1558-66.

17. Rothwell S, Chinoy H, Lamb JA, et al. Focused HLA analysis in Caucasians with myositis identifies significant associations with autoantibody subgroups. *Ann Rheum Dis* 2019; **78**(7): 996-1002.

18. Olerup O, Zetterquist H. HLA-DR typing by PCR amplification with sequence-specific primers (PCR-SSP) in 2 hours: an alternative to serological DR typing in clinical practice including donor-recipient matching in cadaveric transplantation. *Tissue Antigens* 1992; **39**(5): 225-35.

19. Betteridge Z, Tansley S, Shaddick G, et al. Frequency, mutual exclusivity and clinical associations of myositis autoantibodies in a combined European cohort of idiopathic inflammatory myopathy patients. *J Autoimmun* 2019; **101**: 48-55.
