# Supplementary material for: Distinct HLA associations with autoantibody-defined subgroups in idiopathic inflammatory myopathies
Source: eBioMedicine. 2023 Sep 26;96:104804. doi: 10.1016/j.ebiom.2023.104804 (PMC10550566; doi:10.1016/j.ebiom.2023.104804)
Supplement: Supplementary Figure S1 [file mmc3.pdf]

Supplementary Figure 1.

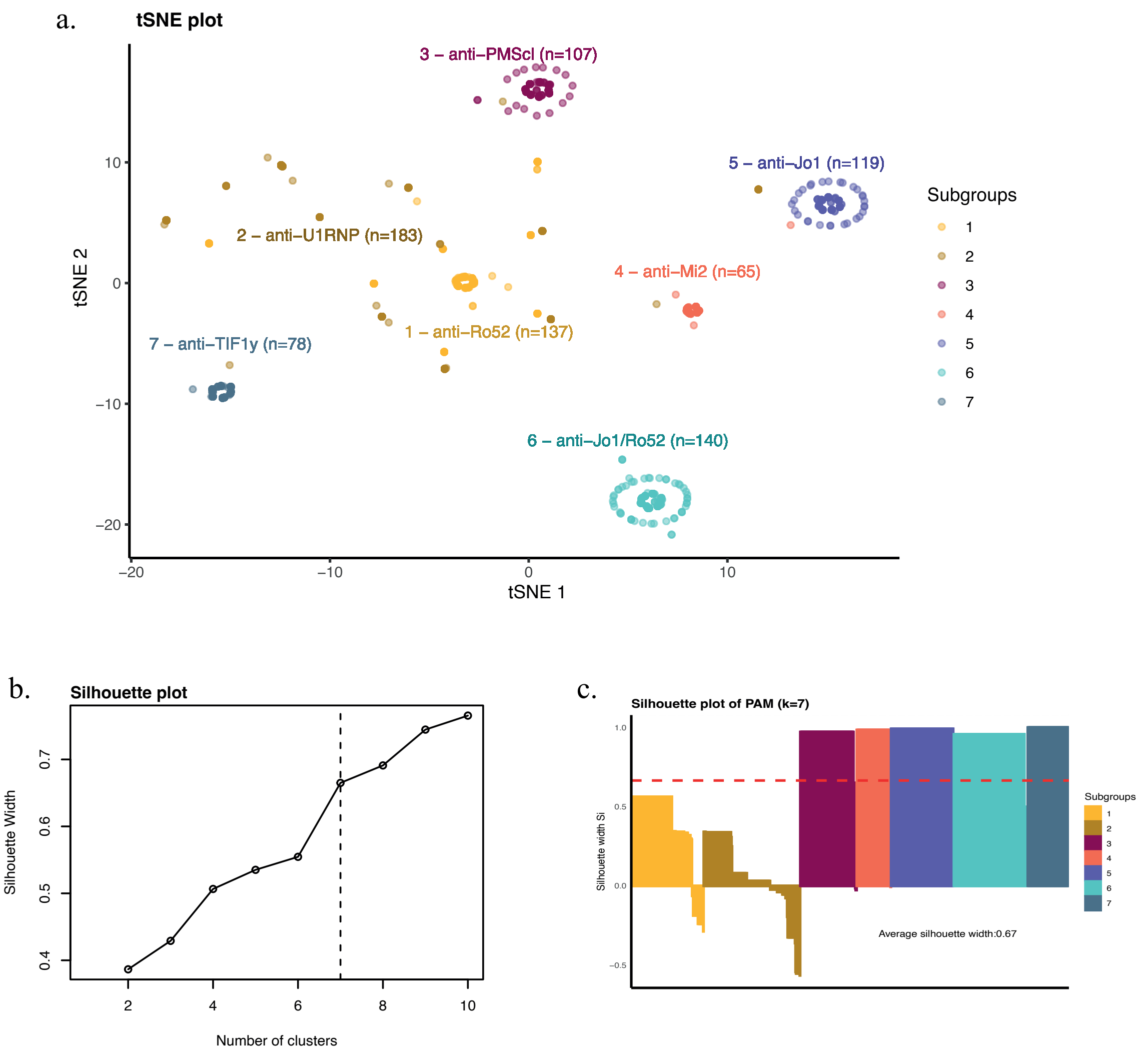

Supplementary Figure 1. tSNE and Silhouette plots representing the subgroups selected.
